# The architecture of kinesin-3 KLP-6 reveals a multilevel-lockdown mechanism for autoinhibition

**DOI:** 10.1038/s41467-022-32048-y

**Published:** 2022-07-25

**Authors:** Wenjuan Wang, Jinqi Ren, Weiye Song, Yong Zhang, Wei Feng

**Affiliations:** 1 National Laboratory of Biomacromolecules, CAS Center for Excellence in Biomacromolecules, Institute of Biophysics, Chinese Academy of Sciences, 15 Datun Road, 100101 Beijing, China; 2 College of Life Sciences, University of Chinese Academy of Sciences, 100049 Beijing, China; 3 Key Laboratory of RNA Biology, CAS Center for Excellence in Biomacromolecules, Institute of Biophysics, Chinese Academy of Sciences, 15 Datun Road, 100101 Beijing, China

**Keywords:** Kinesin, X-ray crystallography, Motor proteins

## Abstract

Autoinhibition of kinesin-3 ensures the proper spatiotemporal control of the motor activity for intracellular transport, but the underlying mechanism remains elusive. Here, we determine the full-length structure of kinesin-3 KLP-6 in a compact self-folded state. Unexpectedly, all the internal coiled-coil segments and domains in KLP-6 cooperate to successively lock down the neck and motor domains. The first coiled-coil segment is melted into several short helices that work with the motor domain to restrain the entire neck domain. The second coiled-coil segment associates with its neighboring FHA and MBS domains and integrates with the tail MATH domain to form a supramodule that synergistically wraps around the motor domain to trap the nucleotide and hinder the microtubule binding. This multilevel-lockdown mechanism for autoinhibition could be applicable to other kinesin-3 motors.

## Abstract (web summary)

Autoinhibition ensures the proper spatiotemporal control of the kinesin-3 motor activity. Here, the authors present the full-length structure of kinesin-3 KLP-6 in an autoinhibited state and observe that all the internal domains entwine around the motor domain via a multilevel-lockdown mechanism.

## Introduction

Kinesins are microtubule-based molecular motors that drive long-range intracellular transport or organize intricate microtubule networks^1–3^. When not transporting cargoes, processive kinesin motors (kinesin-1 to -3) often adopt an autoinhibited conformation to avoid the futile consumption of ATP and the potential blockage of microtubule tracks^4^. Autoinhibition of kinesin motors can also prevent improper cargo association and erroneous movement to ensure the spatiotemporal regulation of the motor activity^5^. As for kinesin-1, autoinhibition regulates its localization and is essential for the dendrite-specific localization of Golgi outposts^6^; a mutation in kinesin-2 that disrupts autoinhibition induces similar defects to the kinesin-2-null mutation during ciliary biogenesis^7,8^; and autoinhibition of kinesin-3 dictates its axonal transport and regulates the size and density of synapses^9,10^. Some disease-related mutations in kinesin-3 are also gain-of-function changes that relieve the autoinhibited state and result in the motor hyperactivation^11,12^.

Kinesin-3 is a unique subfamily of processive kinesin motors in terms of autoinhibition^5^. Instead of forming a constitutive dimer, most of kinesin-3 motors primarily exist as a self-folded monomer for autoinhibition^13,14^, although some kinesin-3 members can adopt an autoinhibited dimeric conformation^15^. In contrast to the tail domain-mediated autoinhibition in kinesin-1 and -2^16–18^, the autoinhibition of kinesin-3 is predominantly mediated by the internal coiled-coil segments and domains^5^. In addition to the N-terminal motor domain (MD) and neck domain (containing the neck linker (NL) and neck coil (NC)), kinesin-3 contains a family-specific forkhead-associated (FHA) domain sandwiched by two coiled-coil segments (coiled-coil 1 (CC1) and CC2) and a characteristic membrane-associated guanylate kinase homolog (MAGUK)-binding stalk (MBS) domain in the middle. In the active state, the FHA domain works together with its preceding coiled-coil segment CC1 to form a stable dimer^19,20^, while the MBS domain can bind to the MAGUK family protein^21^. In the autoinhibited state, the first coiled-coil segment CC1 folds back to associate with the NC and MD to inhibit the NC-mediated dimerization and restrain the neck domain^22,23^, while the second coiled-coil segment CC2 tends to interact with its preceding FHA domain to block the FHA domain-mediated activation of the motor^19,24^. In addition, the MBS domain can bind to the MD to inhibit the microtubule binding^21^. All the above intra-molecular contacts are likely to maintain the motor in a self-folded monomeric conformation^23^. However, it is challenging to conceptualize how these multiple coiled-coil segments and internal domains coordinate to manipulate the self-folding process of full-length kinesin-3 for autoinhibition.

In this study, we determine the full-length structure of the kinesin-3 motor KLP-6 (kinesin-like protein 6 in *Caenorhabditis elegans* for intraflagellar transport in cilia and mitochondrial transport in neurons^25,26^) in a compact self-folded state. Unexpectedly, all the internal coiled-coil segments and domains in KLP-6 cooperate to lock down the neck and motor domains in a successive manner. CC1 is melted into several short helices that associate with the NC and MD to inhibit the entire neck domain, while CC2 binds to both the FHA and MBS domains and integrates with the newly identified MATH domain in the tail region to form a supramodule. This integrated supramodule synergistically associates with the MD to trap the nucleotide and block the microtubule binding. Mutations in the essential interdomain interfaces of self-folded KLP-6 restore the full-length motor activity. This work reveals a multilevel-lockdown mechanism for kinesin-3 autoinhibition.

## Results

### KLP-6 contains all the known inhibitory domains

**Fig. 1:**
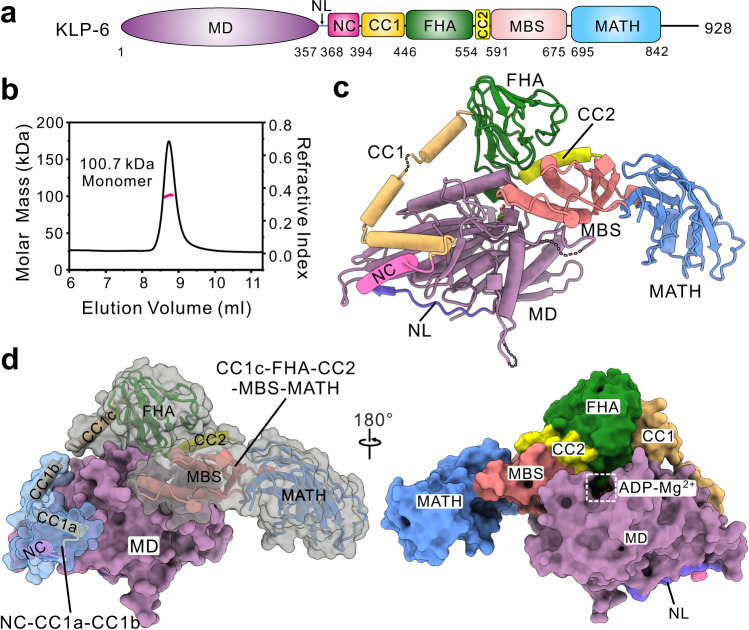
Overall structure of full-length KLP-6. **a** Domain organization of kinesin-3 KLP-6. KLP-6 contains an N-terminal MD, a neck domain (composed of the NL and NC), the CC1-FHA-CC2 tandem followed by a MBS domain, and a newly identified MATH domain. MD motor domain, NL neck linker, NC neck coil, FHA forkhead-associated, CC1 coiled-coil 1, CC2 coiled-coil 2, MBS membrane-associated guanylate kinase homolog (MAGUK)-binding stalk, MATH meprin, and TRAF homology. **b** Biochemical characterization of the oligomeric state of full-length KLP-6 by SEC-MALS. The calculated molecular weight matches a monomeric state. **c** A ribbon diagram of the structure of full-length KLP-6. All the segments and domains are colored with a similar color scheme in **a** for clarity. The unresolved flexible loops in the structure are marked with dashed lines. **d** A combined surface and ribbon diagram of the structure of full-length KLP-6. For binding to the MD in the autoinhibited state, the NC-CC1a-CC1b tandem and the CC1c-FHA-CC2-MBS-MATH supramodule are highlighted.

We previously determined the structure of an N-terminal fragment of KIF13B, including CC1 (the MD-NC-CC1 tandem)^23^, which hardly depict the autoinhibited state of kinesin-3 with other inhibitory domains (such as CC2 and the MBS domain) (Supplementary Fig. 1). We then attempted to work on full-length kinesin-3. However, due to the intrinsic flexibilities between various domains (Supplementary Figs. 1 and 2), it is extremely challenging to obtain the full-length structure of kinesin-3. All the extensive trials of kinesin-3 motors in mammals (such as KIF1A, KIF13A and KIF13B) failed, and we had to resort to those from other different species that contain all the essential domains for autoinhibition. Based on the primary sequence analysis of kinesin-3, KLP-6 from *Caenorhabditis elegans* contains all the known inhibitory domains (including CC1, CC2, and the MBS domain) (Supplementary Fig. 1). Moreover, the internal loops between the various domains of KLP-6 are much shorter and the full-length motor seems to form a more compact conformation (Supplementary Figs. 1 and 2). KLP-6 was therefore chosen as a representative motor for structural dissection of the autoinhibited conformation of full-length kinesin-3 (Fig. 1a).

### The newly identified MATH domain in the tail region of KLP-6

Full-length KLP-6 was expressed using the baculovirus expression system and characterized by the size-exclusion chromatography coupled with multi-angle light-scattering (SEC-MALS) assay. As expected, KLP-6 adopts a stable monomeric state in solution (Fig. 1b). Based on the high quality of the protein sample, we performed the crystal screening. After extensive trials, the crystals of KLP-6 were obtained for structural determination. The structure was determined by the molecular replacement method (using the structures of the MD and the FHA and MBS domains of KIF13B as the searching models) and refined to 3.2 Å (Fig. 1c, Supplementary Fig. 3a, and Supplementary Table 1). In the structure of KLP-6, the extreme N-terminus, a stretch of the C-terminal tail and several internal loops could not be resolved due to their poor electron density maps (Supplementary Fig. 3a, b), and the structure comprises residues from 5 to 842 with the missing of several flexible loops (as indicated by dashed lines in Fig. 1c).

During structural determination, the previously defined coiled-coil segments (the NC, CC1, and CC2) and domains (the MD and the FHA and MBS domains) were well-resolved (Fig. 1c). In addition to these known segments and domains, a new domain in the tail region of KLP-6 was identified based on the electron density map. With the structural homology analysis using the Dali server, this domain adopts a folding topology similar to the meprin and TRAF homology (MATH) domain^27^, i.e., an eight-strained β-sandwich fold with one open end capped by two short helices (Supplementary Fig. 4), and was therefore referred to as the MATH domain. The MATH domain immediately follows the MBS domain in KLP-6 (Fig. 1a, c). To check whether the MATH domain exists in other kinesin-3 motors, we performed the structure-based sequence alignment. Most of the residues for the formation of the MATH domain in KLP-6 are highly conserved in the majority of kinesin-3 motors (such as KIF1A/B and KIF13A/B) (Supplementary Fig. 2), suggesting that this MATH domain could be a structural domain in these kinesin-3 motors.

### Overall structure of full-length KLP-6

In the structure of KLP-6, in addition to the MATH domain, the other domains including the MD and the FHA and MBS domains adopt a canonical structural fold, i.e., the kinesin-motor fold, the β-sandwich-based FHA fold and the α-helix/β-strand-mixed MBS fold (Fig. 1c). In contrast, none of the coiled-coil segments form a canonical coiled-coil dimer, but instead the NC and CC2 adopt a single helical structure and CC1 is melted into three short helices (CC1a to CC1c) (Fig. 1c, d). Unexpectedly, from the overall view of the structure, all the coiled-coil segments and domains of KLP-6 fold back to successively wrap around the MD to form a self-folded compact monomer (Fig. 1d), consistent with the monomeric state in solution (Fig. 1b). Specifically, as previously observed^23^, the CC1a helix folds back to associate with the NC and MD, together with the CC1b helix, to fasten them together, while the CC1c helix packs with the MD and the FHA domain; the CC2 helix interacts with the FHA and MBS domains and integrates them together, and since the MATH domain associates with the MBS domain, the domains following CC1b (from CC1c to MATH) form a compact supramodule (referred to as the CC1c-FHA-CC2-MBS-MATH (CFCMM) supramodule); and the CFCMM supramodule further folds back to synergistically interact with the MD through the FHA and MBS domains, while the MATH domain in this supramodule has little contacts with the MD and protrudes from the central core of the structure (Fig. 1d). Finally, in this compact structure, the NL is docked onto the MD, and ADP and Mg^2+^ are in the nucleotide-binding pocket of the MD (based on their well-resolved electron density maps) (Fig. 1c and Supplementary Fig. 3c).

To probe the potential dynamic properties of the overall conformation, we performed the molecular dynamics simulations of the structure of full-length KLP-6 in solution. Interestingly, during the molecular dynamics simulations, the central core of the full-length structure (including the MD, CC1bc, CC2, and the FHA and MBS domains) is relatively stable without obvious structural changes (Supplementary Fig. 5). In contrast, the NC-CC1a bundle and the MATH domain at the two peripheries of the full-length structure exhibit some conformational changes (Supplementary Fig. 5), indicating that these two peripheral parts are somewhat dynamic in solution.

### Interdomain interfaces in self-folded KLP-6

**Fig. 2:**
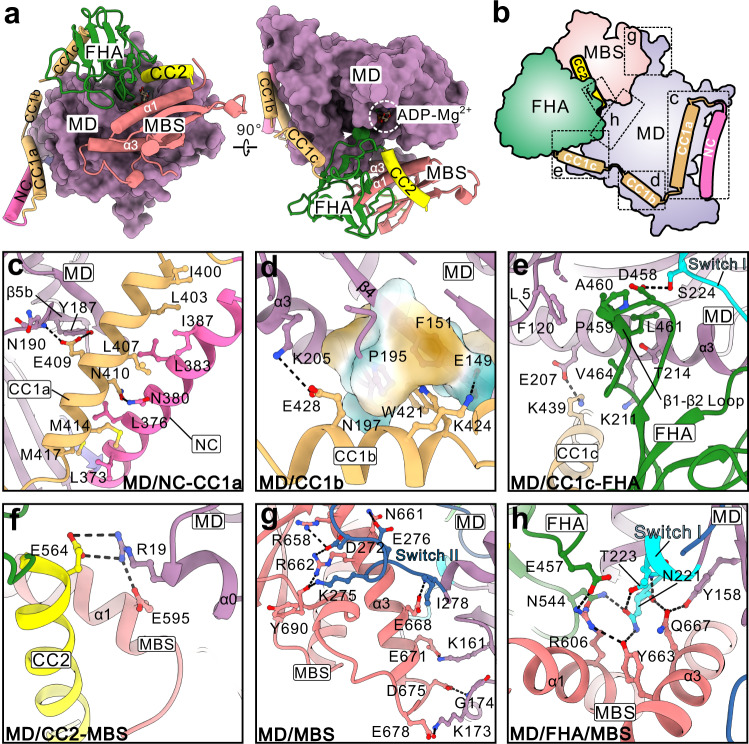
Interdomain interfaces in self-folded KLP-6. **a** A combined surface and ribbon representation showing the MD-involved interdomain interfaces. The MD is in the surface representation, and the other domains are in the ribbon representation. The MATH domain is removed due to no contact with the MD. **b** A schematic model illustrates the MD-involved interdomain interfaces that are divided into six different sites. **c**–**h** A combined ribbon-and-stick model showing the MD-involved interdomain interfaces in the MD/NC-CC1a (**c**), MD/CC1b (**d**), MD/CC1c-FHA (**e**), MD/CC2-MBS (**f**), MD/MBS (**g**), and MD/FHA/MBS (**h**) sites. The color scheme follows that in Fig. 1c. The sidechains of the residues in the interdomain interfaces are shown as sticks. Switch I and II of the MD are highlighted and colored in cyan and dark blue, respectively.

Since the coiled-coil segments (from the NC to CC2) and structural domains (except for the MATH domain) wrap around the MD (Fig. 2a), the interdomain interfaces in self-folded KLP-6 are categorized into the MD-involved and non-MD-involved interfaces. The MD-involved interfaces can be further divided into six different sites (Fig. 2b). In the MD/NC-CC1a site, CC1a and the NC associates anti-parallelly with a packing network formed by the hydrophobic residues from the two helices, N410 from CC1a forms a hydrogen bond with N380 from the NC, and E409 from CC1a makes the hydrogen-bonding contacts with Y187 and the backbone of N190 from the MD (Fig. 2c). In the MD/CC1b site, bulky W421 from CC1b inserts into a small pocket in the MD, and K424 and E428 from CC1b form the electrostatic interactions with E149 and K205 from the MD, respectively, to seal this pocket (Fig. 2d). In the MD/CC1c-FHA site, the β1–β2 loop of the FHA domain packs into a large pocket in the MD (Fig. 2a), i.e., D458 from the FHA domain forms a hydrogen bond with S224 from Switch I of the MD, P459, A460, L461, and V464 from the FHA domain make the hydrophobic contacts with F120, K211 and T214 from the MD, and K439 from CC1c forms the electrostatic interaction with E207 from the MD (Fig. 2e). In the MD/CC2-MBS site, E564 from CC2 and E595 from the MBS domain form the electrostatic interactions with R19 from the MD (Fig. 2f). In the MD/MBS site, the MBS domain associates with the MD through two electrostatic/hydrogen-bonding networks, i.e., R658, N661, R662 and the backbone of Y690 from the MBS domain form one interaction network with D272, K275, and E276 from Switch II of the MD, E671, D675, and E678 from the MBS domain form the other one with K161, K173, and the backbone of G174 from the MD, and between the two networks, E668 from the MBS domain forms an additional hydrogen bond with the backbone of I278 from the MD (Fig. 2g). In the MD/FHA/MBS site, E457 and N544 from the FHA domain, R606, Y663, and Q667 from the MBS domain, and Y158, N221, and T223 from the MD form an extensive electrostatic/hydrogen-bonding network in the interdomain packing core (Fig. 2b, h).

The non-MD-involved interfaces are responsible for the CFCMM supramodule formation and can be divided into three different sites (Supplementary Fig. 6a). In the CC1c-FHA site, M442, K445, and K446 from CC1c form the hydrophobic contacts with V466 and F468 from the FHA domain (Supplementary Fig. 6b). In the FHA-CC2-MBS site, bulky Y563 from CC2 anchors into a shallow pocket in the FHA domain (capped by Q567 from CC2), I570 and A571 from CC2 make the hydrophobic contacts with F521 and F540 from the FHA domain and L597, M598 and L601 from the MBS domain, and E569 from CC2 makes the electrostatic interaction with R538 from the FHA domain (Supplementary Fig. 6c). In the MBS-MATH site, the MBS domain associates with the MATH domain through a hydrophobic packing core between the two domains, and the backbones of E648, D649 and Y651 from the MBS domain form an additional hydrogen-bonding network with Q838 and R839 from the MATH domain (Supplementary Fig. 6d, e). Taken together, the extensive hydrophobic, electrostatic, and hydrogen-bonding interactions between the interdomain interfaces integrate all the segments and domains together to assemble a compact structure.

### CC1-mediated lockdown of the entire neck domain

**Fig. 3:**
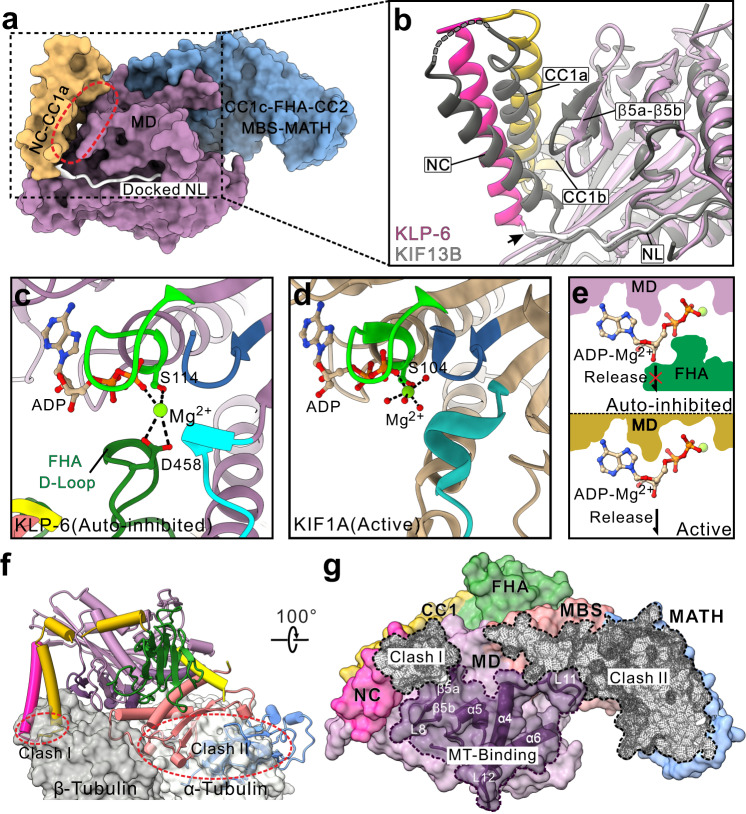
Multilevel lockdown of KLP-6 for autoinhibition. **a**, **b** CC1-mediated lockdown of the neck domain. A combined surface and ribbon representation showing that the NL is locked in a docked state on the MD (**a**). Structural comparison of the N-terminal fragment of KLP-6 with that of KIF13B (**b**). In this comparison, the MD-NC-CC1 tandem of KIF13B (PDB code: 6A20) is colored in gray and the similar fragment of KLP-6 is colored as panel **a**. The NL of KLP-6 is one-residue longer than that of KIF13B (indicate by a black arrow). **c**–**e** The D-loop of the FHA domain traps ADP-Mg^2+^ in the nucleotide-binding pocket. A close-up view of the nucleotide-binding pocket in the MD of KLP-6 (**c**) and that of KIF1A (PDB code: 1I5S) (**d**). ADP are shown as sticks and Mg^2+^ are shown in spheres. The interactions with Mg^2+^ in the nucleotide-binding pocket are highlighted by dashed lines. The P-loop and switch I/II of the MD are colored in lemon, cyan and blue, respectively. Compared with the active MD of KIF1A, the signature residue D458 from the D-loop of the FHA domain in KLP-6 interacts with Mg^2+^ and traps ADP-Mg^2+^ in the nucleotide-binding pocket to prevent the nucleotide exchange (**e**). **f**, **g** Structural superimposition showing the MBS-MATH-mediated steric hindrance of the microtubule binding for KLP-6. The structure of full-length KLP-6 is superimposed with that of the kinesin-1/tubulin complex (PDB code: 4HNA) (**f**). The microtubule-binding site of the MD and the two steric clashes in KLP-6 for binding to microtubules are indicated by the surface areas with dashed lines (**g**). In addition to the prominent clashes between the MBS-MATH tandem and α-tubulin, the NC-CC1a bundle also exhibits certain steric conflicts with β-tubulin.

CC1 was previously identified as the key inhibitory segment in kinesin-3 to block the neck domain (that is composed of the NL and NC)^22,23^. In the structure of KLP-6, CC1 is melted into several short helices that associate with the NC and MD (Fig. 2b). The association of CC1 with the NC would prevent the NC dimer formation, and the CC1-mediated further tethering of the NC to the MD could restrain the motion of the NC and lock the NL in a docked state on the MD (Figs. 2b and 3a). Given that the NC dimer formation and NL docking/undocking (with the MD) are essential for processive movement^28,29^, CC1-mediated fastening of the NC and MD can lock down the entire neck domain of KLP-6 (Fig. 3a). The similar MD-aided lockdown of the neck domain was demonstrated in our studies of KIF13B using the MD-NC-CC1 tandem^23^, supporting that this CC1-mediated inhibitory mode may be employed by most of kinesin-3 motors. Consistently, most of the essential residues in CC1 for the interdomain packing interfaces are highly conserved in these kinesin-3 motors (Supplementary Fig. 2).

Based on the structural analysis, the MD-NC-CC1ab tandem is likely to form a sub-complex with the interdomain interactions. To probe the potential formation of this sub-complex, we first performed the molecular dynamics simulations of the MD-NC-CC1ab tandem in solution. Consistent with the full-length structure of KLP-6, the overall structure of the MD-NC-CC1ab tandem is also relatively stable (but with certain structural changes in the NC-CC1a bundle) and seems to be more stable without the account of the NC-CC1a bundle (Supplementary Fig. 7a), suggesting the tendency of the sub-complex formation. Next, we biochemically characterized the stability of the MD-NC-CC1ab tandem by the thermal denaturation assay. We made the point mutations (N410A and W421A) in the interdomain interfaces to dissociate the sub-complex. As expected, the MD-NC-CC1ab tandem is relatively stable with a high denaturation temperature Tm (characterized by the temperature at the half of full denaturation) (Supplementary Fig. 7b). Moreover, the denaturation temperature Tm could be decreased by the point mutations in the interdomain interfaces (Supplementary Fig. 7b), supporting that these interdomain interfaces are essential for the formation of the sub-complex.

Given the similar inhibition mode in KLP-6 and KIF13B, we compared the structures of their N-terminal fragments (containing CC1a and CC1b) in detail (Fig. 3b). As expected, the two structures could be well superimposed (with the RMSD of ~0.91 Å for backbone atoms) and exhibit the similar packing of CC1a and CC1b to the NC and MD (Fig. 3b). However, relative to the MD, the NC helix possesses some local differences between the two structures, i.e., this helix in KLP-6 moves a little bit away from the MD (Fig. 3b). With the account of the beginning of the NC, the NL in KLP-6 is one-residue longer than that in KIF13B. The extended NL in KLP-6 would somewhat push the NC helix away from the MD and thus might cause the slight structural differences of this helix (Fig. 3b).

### Integration of the CFCMM supramodule by CC2 for autoinhibition

In contrast to CC1a and CC1b, CC1c is not involved in the inhibition of the neck domain but associates with the FHA domain to stabilize the contacts between the FHA domain and the MD (Fig. 2b, e), demonstrating that the three short helices of CC1 in KLP-6 exhibit the distinct functions for autoinhibition. Similar to CC1c, CC2 forms a single helix that folds back to interact with the FHA domain and the two helices are likely to clamp the FHA domain onto the MD (Fig. 2b). More intriguingly, CC2 also binds to its following MBS domain to bridge the FHA and MBS domains together and drive the further self-folding of the MBS domain toward the MD (Fig. 2b), supporting that, in addition to CC1, CC2 is another essential segment in KLP-6 to organize the intra-molecular interaction network. Since the MATH domain associates with the MBS domain (Supplementary Fig. 6d, e), with the aid of the central CC2 helix, the segments and domains from the middle to the tail of KLP-6 are integrated together to form the CFCMM supramodule for autoinhibition.

Similar to the MD-NC-CC1ab tandem, the CFCMM supramodule seems to form another sub-complex with the interdomain interactions. We performed the same set of molecular dynamics simulations for the CFCMM supramodule and found that the overall structure of the CFCMM supramodule is relatively stable in solution and becomes more stable without the account of the MATH domain (Supplementary Fig. 7c), consistent with the full-length structure of KLP-6 (Supplementary Fig. 5). We also characterized the stabilities of the CFCMM supramodule and its mutants (with the I570Q and F698Q mutations in the interdomain interfaces) by the thermal denaturation assay. As expected, the CFCMM supramodule is relatively stable in solution with a high denaturation temperature Tm, which could be decreased by the point mutations in the interdomain interfaces (Supplementary Fig. 7d), thus also supporting the tendency of the sub-complex formation and the essential roles of these interdomain interfaces.

CC2 was demonstrated to associate with the FHA domain in KIF1A^24^, but the mechanism remains to be determined. In the structure of KLP-6, in addition to packing with the FHA domain, CC2 unexpectedly associates with the MBS domain and sticks the two domains together for binding to the MD (Supplementary Fig. 8a), suggesting that, instead of the proposed sole role for the FHA domain, CC2 is a more versatile segment for dictating the assembly of the CFCMM supramodule. Since the CC1-FHA tandem in kinesin-3 can form a stable dimer for the motor dimerization and activation^19,20^, we next compared the structure of the CFCMM supramodule with that of the CC1-FHA dimer from KIF13A (Supplementary Fig. 8b). Upon superimposition of the FHA domain, the other domains in the CFCMM supramodule impacts the dimer interface (Supplementary Fig. 8b), indicating that this supramodule would prevent the CC1-FHA dimer formation, in accordance with the negative role of the CC2/FHA binding for the motor dimerization^24^. However, CC2 in KLP-6 seems to be much shorter than that in other kinesin-3 motors (Supplementary Figs. 1 and 2), and it is possible that extended CC2 may exhibit more functions than the integration of the CFCMM supramodule.

### A unique loop of the FHA domain traps ADP-Mg^2+^ in the nucleotide-binding pocket

With the formation of the CFCMM supramodule, the FHA domain associates with the MD in the structure of KLP-6, and the β1-β2 loop of the FHA domain inserts into a tailored pocket in the MD nearby the nucleotide-binding pocket (in which ADP and Mg^2+^ are located) (Fig. 2a). In addition to contacting with Switch I of the MD (Fig. 2e), the signature residue D458 from this β1-β2 loop (referred to as the D-loop) makes the electrostatic interactions with Mg^2+^, together with the phosphate group of ADP and a serine from the P-loop of the MD, to stably chelate this ion in the nucleotide-binding pocket (Fig. 3c). For kinesins, Mg^2+^ is the critical bivalent ion that coordinates and stabilizes the nucleotide for binding to the P-loop of the MD (Fig. 3d), and this ion should be released from the nucleotide-binding pocket for the nucleotide exchange (i.e., the ADP release and ATP binding). The direct binding of the D-loop of the FHA domain to Mg^2+^ would stabilize it in the nucleotide-binding pocket and block its release channel (Fig. 3e). Since Mg^2+^ always works together with ADP in the nucleotide-binding pocket, the D-loop of the FHA domain resembles a big plug to trap ADP-Mg^2+^ in this pocket and inhibit the nucleotide exchange (Fig. 3e).

Based on the structural conservation analysis of KLP-6, the D-loop of the FHA domain is highly conserved in most of kinesin-3 motors (Supplementary Fig. 9a, b), suggesting that the interaction between Mg^2+^ and the D-loop of the FHA domain may exist in other kinesin-3 motors. We further analyzed the distance between Mg^2+^ and the D-loop of the FHA domain during the molecular dynamics simulations of full-length KLP-6. Interestingly, there are two populations of the Mg^2+^/D-loop distance, i.e., the major population (~70%) with an average distance of ~4 Å and the minor population (~30%) with an average distance of ~6 Å (Supplementary Fig. 9c). The close distance between Mg^2+^ and the D-loop (~4-6 Å) during the molecular dynamics simulations demonstrates the constant and stable binding between the two components albeit with somewhat dynamic properties. Thus, it seems that ADP-Mg^2+^ in the nucleotide-binding pocket is unlikely to be easily released due to the stable interaction with the D-loop of the FHA domain.

The FHA domain in KIF1A was suggested to associate with the MD albeit with the lack of evidence^24^. The structure of KLP-6 demonstrated the direct binding of the FHA domain to the MD (Fig. 2e). The trapping of ADP-Mg^2+^ within the MD by the FHA domain is unexpected and of significance for autoinhibition (Fig. 3c). Given the higher concentration of ATP than that of ADP at the cellular level, this ADP-Mg^2+^-trapped mechanism would completely block the competition of ATP with ADP and avoid the nucleotide-exchange process for the futile consumption of ATP (Fig. 3e). More intriguingly, the essential residues in the D-loop of the FHA domain are highly conserved in the majority of kinesin-3 motors (Supplementary Figs. 2 and 9b), indicating that this FHA-mediated trapping of ADP-Mg^2+^ would happen in these kinesin-3 motors. Finally, since the FHA domain was demonstrated to be essential for the motor dimerization^19^, the association with and inhibition of the MD in self-folded KLP-6 would also suggest that this domain could resemble a double-edged sword for the control of both motor inhibition and activation.

### MBS-MATH-mediated steric hindrance of the microtubule binding

In addition to the FHA domain, the MBS domain in the CFCMM supramodule associates with the MD as well, and the α3 helix of the MBS domain packs with Switch I/II of the MD (Fig. 2g, h). In the MD, Switch II is neighboring to the microtubule-binding site for coupling the binding of microtubules with the ATPase activity of the MD (Supplementary Fig. 10a). The association of the MBS domain to Switch II would somewhat block this coupling and interfere with the microtubule-binding site of the MD. To assess this possibility, we compared the structure of KLP-6 with that of the kinesin-1/tubulin complex (Supplementary Fig. 10a). Upon superimposition of the MD, both the MBS and MATH domains in self-folded KLP-6 possess the steric clashes with α-tubulin (Fig. 3f, g), suggesting that the MBS-MATH tandem could sterically hinder the microtubule-binding capacity. Although the MATH domain was previously unknown, the similar inhibitory role of the MBS domain for the MD was found in KIF13B^21^, indicating that this domain could be dedicated for blocking the binding of microtubules. More intriguingly, the NC-CC1a bundle also exhibits certain steric conflicts with β-tubulin (Fig. 3f, g), which may suggest the additional negative effect of this two-helix bundle on the microtubule binding.

The MBS domain in KIF13B was reported to bind to the guanylate kinase (GK) domain of the MAGUK family protein discs large homolog (DLG) for the release of the autoinhibited conformation^21^. To evaluate the potential impact of the binding of the GK domain for the motor activation, we compared the structure of KLP-6 with that of the MBS/GK complex formed between KIF13B and DLG (Supplementary Fig. 10b). In KLP-6, most of the GK-binding site of the MBS domain is exposed and accessible (Supplementary Fig. 10b), indicating that the GK domain is likely to be capable of binding to the MBS domain in the autoinhibited state. When the MBS domains from the two structures are superimposed, some structural clashes between the GK domain and Switch II of the MD appear (Supplementary Fig. 10b), suggesting that the binding of the GK domain would somewhat impact the autoinhibited conformation of KLP-6 and thus might contribute to the motor activation.

### Evaluation of the interdomain interfaces for the full-length motor inhibition

**Fig. 4:**
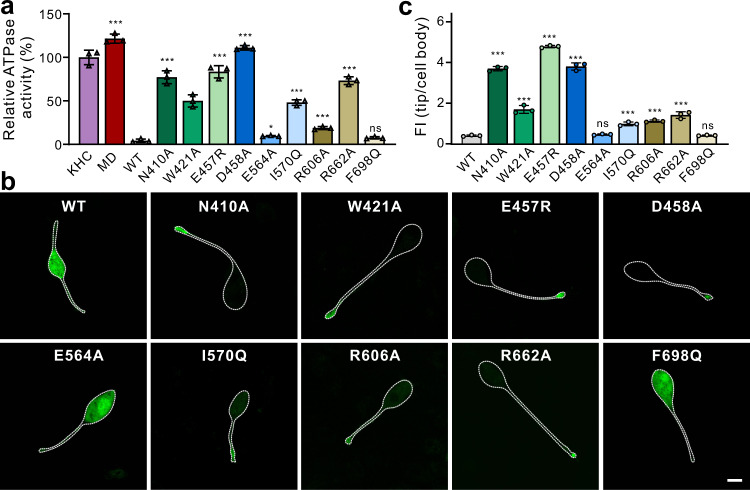
Disruptions of the interdomain interfaces restore the motor activity of KLP-6. **a** Microtubule-stimulated ATPase activities of the wild-type and various mutants of full-length KLP-6. Data were normalized by the microtubule-stimulated ATPase activity of KHC as 100%. Each protein sample had two replicates and each measurement was repeated three times independently. Each bar represents the mean value ± SD. ^***^P < 0.001; ^*^P < 0.05; ns, no significant difference, unpaired, two-tailed Student’s *t* test. Source data are provided as a Source Data file. **b** Cellular localizations of full-length KLP-6 and its various mutants in the N2A cells. Wild-type KLP-6 was largely localized in the cell body, while most of the mutants showed significantly increased accumulations at the cell tips (except for the E564A and F698Q mutants). Each experiment was repeated three times independently. Scale bar: 10 μm. **c** Quantification of the cellular distribution data shown in panel **b**. The ratio of the tip to cell body average fluorescence intensity (FI) was quantified for each construct. Each experiment was repeated three times independently (i.e., three datasets), and in each dataset, the data were collected for 15 cells and the average result was used to represent each experiment. Each bar represents the mean value ± SD. ^***^P < 0.001; ns, no significant difference, unpaired, two-tailed Student’s *t* test. Source data are provided as a Source Data file.

Based on the structural analysis, the internal segments and domains (from the middle to tail) in KLP-6 are all involved in assembling the compact structure to lock down the full-length motor (Figs. 2 and 3). To evaluate the essential roles of the interdomain interfaces in this self-folded conformation for KLP-6 autoinhibition, we made the point mutations in different interdomain interfaces (N410A in MD/NC-CC1a, W421A in MD/CC1b, D458A in MD/CC1c-FHA, E564A in MD/CC2-MBS, R662A in MD/MBS, E457R and R606A in MD/FHA/MBS, I570Q in FHA-CC2-MBS, and F698Q in MBS-MATH) and checked the effects of these mutations on the microtubule-stimulated ATPase activity of the full-length motor (Fig. 4a). Without adding microtubules, the basal ATPase activities of all the KLP-6 proteins were low (Supplementary Fig. 11). As the control, the MD alone exhibited the high microtubule-stimulated ATPase activity, while the activity of wild-type KLP-6 was low, consistent with the autoinhibited state of the full-length motor (Fig. 4a). As expected, most of the point mutations that disrupt the interdomain interfaces could significantly restore the microtubule-stimulated ATPase activity of the full-length motor (Fig. 4a). In contrast, the E564A mutation in the MD/CC2-MBS interface only slightly restored the activity and the F698Q mutation in the MBS-MATH interface had little impact (Fig. 4a), suggesting that these interdomain interfaces may have minor contributions to autoinhibition (see “Discussion” below). To assess the impacts of the mutations on KLP-6 in cellulo, we further checked the activity of the full-length motor by using the cell-based assay (Fig. 4b). Consistent with the in vitro ATPase activity assay, wild-type KLP-6 was largely localized in the cell body, while most of the mutants were enriched at the cell periphery (except for the E564A and F698Q mutants) (Fig. 4b, c), suggesting that the cellular activity of the full-length motor was largely restored by these point mutations in the interdomain interfaces. Taken together, the interdomain interfaces (except for the MD/CC2-MBS and MBS-MATH interfaces) for the formation of self-folded KLP-6 are essential for full-length motor inhibition.

## Discussion

In most of kinesin-3 motors, autoinhibition is controlled by the internal coiled-coil segments and domains^5^. In this work, we report the full-length structure of the kinesin-3 motor KLP-6 in an autoinhibited state (Fig. 1c), which reveals a number of previously unknown features that contribute to kinesin-3 autoinhibition. First, as the key inhibitory segment, CC1 can be broken into three short helices (more than two helices in our previous studies) that not only associate with the NC and MD but also interact with the FHA domain to facilitate the formation of the self-folded motor (Fig. 1d). Second, in addition to packing with the FHA domain, CC2 is further capable of binding to the MBS domain to stick the two domains together for synergistically associating with the MD (Fig. 2a, b). Third, the unique D-loop of the FHA domain is unexpected to interact with Mg^2+^ and trap ADP-Mg^2+^ in the nucleotide-binding pocket of the MD (Fig. 3c). Finally, the MATH domain is a newly identified domain that immediately follows and associates with the MBS domain to block the microtubule-binding capacity of the MD (Fig. 3f, g). These unexpected features derived from the full-length structure of KLP-6 expand the current knowledge for kinesin-3 autoinhibition.

The MATH domain was originally identified in the meprin and TRAF family proteins and contributes to the tetramerization of meprin and the trimerization of TRAF^30^, indicating that this domain primarily functions as an oligomerization domain. Thus, upon the release of autoinhibition, it is possible that the MATH domain in kinesin-3 could mediate the dimerization/oligomerization of the motor. In addition, in the full-length structure of KLP-6, the MATH domain associates with the MBS domain that can bind to cargo adaptors (Supplementary Fig. 6), suggesting that this domain might also function as a structural domain for recognizing cargo adaptors. However, the mutation in the MBS-MATH interface had little impact on the autoinhibition (Fig. 4). The possible explanation could be that the dissociation of the MATH domain (with the mutation in the MBS-MATH interface) does not touch the FHA-CC2-MBS-mediated central packing core for autoinhibition, consistent with the dynamic properties of this peripheral domain during the molecular dynamics simulations (Supplementary Fig. 5). On the other hand, the mutation in the MD/CC2-MBS interface slightly impacted on the autoinhibition (Fig. 4). In comparison to other sophisticated MD-involved interfaces, the MD/CC2-MBS interface is composed of a small charge-interaction network formed by only three charged residues (Fig. 2), which may be also consistent with the minor role of this interdomain interface for autoinhibition.

**Fig. 5:**
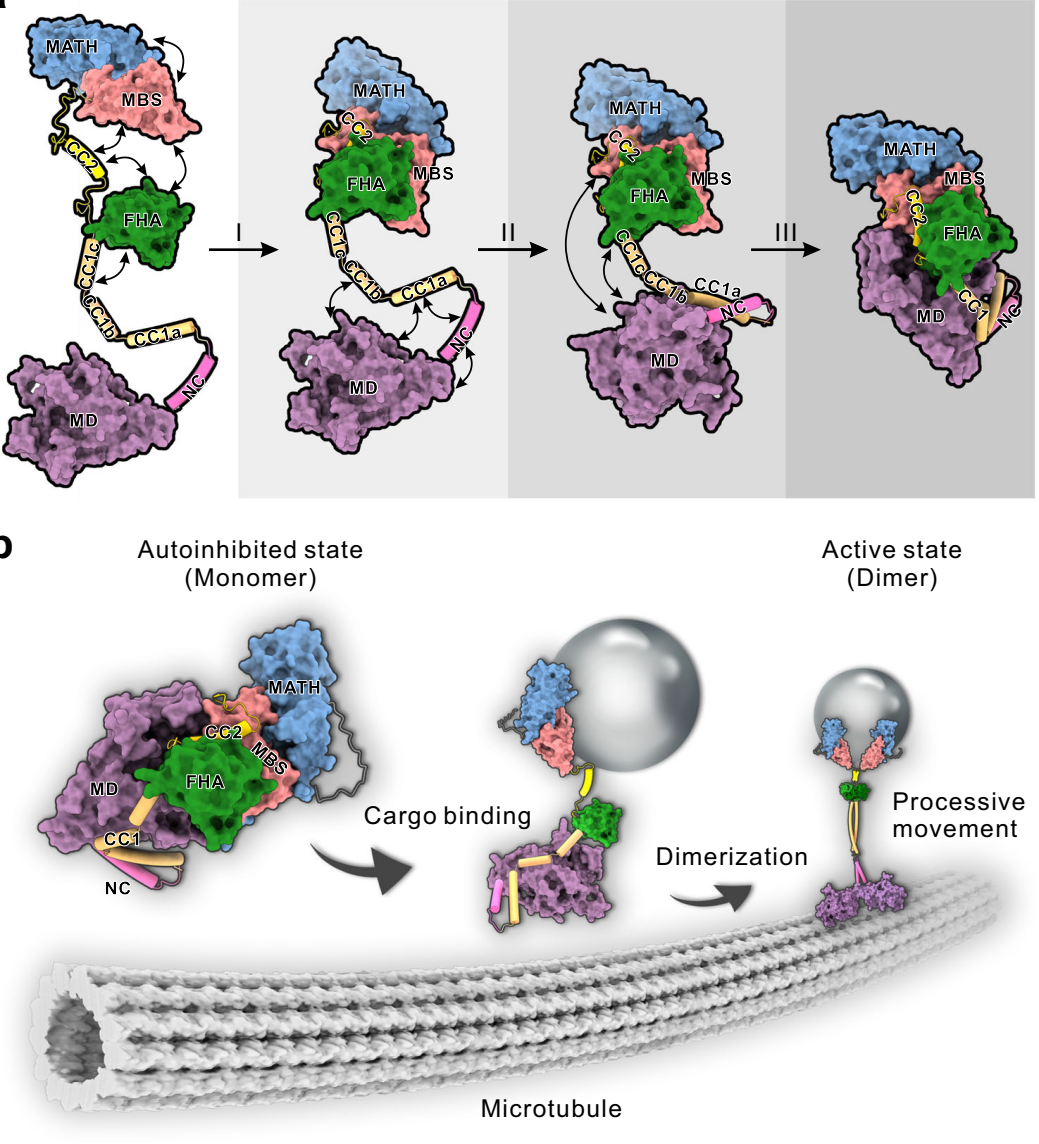
Working models for the multilevel autoinhibition and the cargo-mediated activation of kinesin-3 KLP-6. **a** A working model for the cooperative self-folding process of full-length KLP-6 for autoinhibition with multiple levels. In level I, CC2 associates with the FHA and MBS domains and cooperates with CC1c and the MATH domain to form the CFCMM supramodule; in level II, CC1 (CC1a and CC1b) folds back to pack with the NC and MD to restrain the neck domain; and in level III, the CFCMM supramodule folds back to associate with the MD to form a compact self-folded motor. In this model, the MD and the FHA, MBS and MATH domains are in the surface representation, and the NC, CC1, and CC2 are in the ribbon representation. **b** A schematic working model for the cargo-mediated activation of kinesin-3 KLP-6. In the autoinhibited state, KLP-6 adopts a compact self-folded conformation and all the internal coiled-coil segments and domains cooperate to lock down the neck and motor domains (left panel). Upon binding to cargoes or cargo adaptors likely through the MBS (and/or MATH) domain, the CFCMM supramodule would be disassembled to relieve the inhibitions of the FHA and motor domains (middle panel). The formation of the CC1-FHA dimer would further release the CC1-mediated inhibition of the neck domain, and the NC, CC1, and the FHA domain would work together to promote the formation of a dimeric motor for processive movement (right panel).

Based on the full-length structure, the formation of autoinhibited KLP-6 resembles the self-folding process that need the cooperation between different domains and can be roughly divided into three levels (level I–III) for clarity (Fig. 5a), which might be supported by the potential formation of the two sub-complexes (Supplementary Fig. 7). In level I, since the FHA domain is the key domain to control the motor dimerization, CC2 would associate with the FHA and MBS domains and cooperate with CC1c and the MATH domain to form the CFCMM supramodule that blocks the FHA domain-mediated dimerization (Fig. 5a). In level II, melted CC1 would fold back to pack with the NC and work with the MD to restrain the entire neck domain (including the NC and NL) (Fig. 5a). In level III, the CFCMM supramodule would fold back to associate with the MD to trap the nucleotide in the nucleotide-binding pocket and block the microtubule binding with the cooperation of the FHA, MBS and MATH domains (Fig. 5a). Thus, KLP-6 undergoes a cooperative self-folding process for the multilevel lockdown of the full-length motor. Moreover, it is possible that the three proposed levels could also happen simultaneously and cooperate with each other for autoinhibition.

The cooperative lockdown with multiple levels would possess some advantages for the cargo-mediated dimerization and activation of the full-length motor, which was demonstrated in the majority of kinesin-3 motors^14,31^. Due to the potential cooperation between the different inhibitory domains, the disturbance of one of them by cargo binding would impact the self-folding process to release the autoinhibited state (Fig. 5b). Consistently, mutations in the inhibitory segment or domain in KLP-6 could restore the full-length motor activity (Fig. 4). The potential correlation between the different levels of lockdown would also facilitate the cargo-mediated activation, e.g., the dual roles of the FHA domain for the motor inhibition (in level III) and dimerization (inhibited in level I) could be switched by the binding of cargoes to successively release the motor and neck domains (Fig. 5a, b). Upon binding to cargoes or cargo adaptors likely through the MBS domain, the CFCMM supramodule would be disassembled to relieve the inhibitions of the FHA and motor domains (Fig. 5b). The formation of the CC1-FHA dimer would then release the CC1-mediated inhibition of the neck domain and promote the formation of the motor dimer (likely through a chain reaction of the coiled-coil segments) for processive movement (Fig. 5b).

In addition to the cargo-mediated dimerization and activation, the recent study of the kinesin-3 motor KIF1C demonstrated that KIF1C adopts an autoinhibited dimer through the motor-stalk interaction and the binding of cargo adaptors to the stalk region can release the autoinhibited conformation^15^. In accordance with this dimeric autoinhibitory mode, based on the domain organization of KIF1C, some structural domains in most of kinesin-3 members (such as the MBS and MATH domains) that mediate the formation of the inactive monomer are missing in KIF1C (Supplementary Fig. 1), but instead KIF1C contains the coiled-coil segments in the tail region that may mediate the dimeric conformation of the motor in the autoinhibited state.

Finally, autoinhibition of kinesin-3 is essential for the proper control of motor activity and the deviation would lead to severe diseases^11^. Based on the sequence alignment of KIF1A and KLP-6, the disease-related mutations in KIF1A were mapped onto the structure of KLP-6. Interestingly, some mutations happen to be located in the interdomain interfaces in the self-folded compact structure of KLP-6 (Supplementary Fig. 12), suggesting that these disease-related mutations would impact the autoinhibited conformation and thus may cause motor hyperactivation.

## Methods

### Protein expression and purification

DNA sequences encoding *Caenorhabditis elegans* KLP-6 and various mutants were each cloned into a modified pFastBac1 (Gibco, 10359016) vector which contains a C-terminal GFP-His_6_-Strep tandem tag. The mutations in KLP-6 were generated by using the standard PCR-based method and confirmed by DNA sequencing. All the primers used in the study were summarized in Supplementary Table 2. The wild-type KLP-6 and various mutants were expressed in insect sf9 cells (Gibco, 11496015) using the Bac-to-Bac baculovirus expression system (Gibco, 10359016). Briefly, the pFastBac1 plasmids were transformed into *Escherichia coli* DH10Bac cells (Gibco, 10359016) to acquire the bacmids. The P1 baculoviruses were obtained by transfecting the bacmids into sf9 cells using the Cellfectin II reagent (Gibco, 10362100), and the P2 baculoviruses were collected after 72 h of amplification from the P1 baculoviruses. All the plasmids and strains used in the study were summarized in Supplementary Table 3. For the large-scale production of proteins, sf9 cells were cultured to a density of 2 × 10^6^ cells/ml at 28 °C, and then the P2 baculoviruses were added at a ratio of 1% (v/v) to initiate transfection. After the 72-h cultivation, the cells were harvested and suspended in the buffer containing 50 mM Tris-HCl, pH 8.0, 500 mM (NH_4_)_2_SO_4_, 1 mM MgCl_2_, 1 mM EGTA, 5 mM imidazole. The proteins were purified by Ni^2+^-Sepharose 6 Fast Flow (GE healthcare, 17-5318-03) affinity chromatography with the washing buffer (50 mM Tris-HCl, pH 8.0, 500 mM (NH_4_)_2_SO_4_, 1 mM MgCl_2_, 1 mM EGTA, 25 mM imidazole) and the elution buffer (25 mM HEPES, pH 7.5, 150 mM NaCl, 1 mM MgCl_2_, 1 mM EGTA, 500 mM imidazole). The extra affinity purification was applied using the Strep-Tactin Sepharose resin (IBA Lifesciences, 2-1201-025) with the buffer (25 mM HEPES, pH 7.5, 150 mM NaCl, 1 mM MgCl_2_, 1 mM EGTA). The proteins were eluted in the buffer (25 mM HEPES, pH 7.5, 150 mM NaCl, 1 mM MgCl_2_, 1 mM EGTA, 100 mM biotin) and were further purified by size-exclusion chromatography (Superdex-200 10/300, GE healthcare) with the buffer containing 25 mM HEPES, pH 7.5, 150 mM NaCl, 1 mM MgCl_2_, 1 mM EGTA, 1 mM DTT. For wild-type KLP-6, 0.1 mM ADP was supplemented in the buffer for crystallization.

DNA sequences encoding the MD-NC-CC1ab tandem and the CFCMM supramodule of *Caenorhabditis elegans* KLP-6 and various mutants were each cloned into a modified pET32a vector. The resulting constructs contained an N-terminal His_6_-tag. All the KLP-6 fragments were expressed in *Escherichia coli* BL21 (codon plus) host cells at 16 °C. For the MD-NC-CC1ab tandem and its mutants, the cells were harvested and suspended in the buffer containing 50 mM Tris-HCl, pH 8.0, 500 mM (NH_4_)_2_SO_4_, 1 mM MgCl_2_, 1 mM EGTA, 5 mM imidazole. The proteins were purified by Ni^2+^-Sepharose 6 Fast Flow (GE healthcare, 17-5318-03) affinity chromatography with the washing buffer (50 mM Tris-HCl, pH 8.0, 500 mM (NH_4_)_2_SO_4_, 1 mM MgCl_2_, 1 mM EGTA, 25 mM imidazole) and the elution buffer (50 mM Tris-HCl, pH 8.0, 500 mM (NH_4_)_2_SO_4_, 1 mM MgCl_2_, 1 mM EGTA, 500 mM imidazole). For the CFCMM supramodule and its mutants, the cells were harvested and suspended in the buffer containing 50 mM Tris-HCl, pH 8.0, 500 mM NaCl, 5 mM imidazole. The proteins were purified by Ni^2+^-Sepharose 6 Fast Flow (GE healthcare, 17-5318-03) affinity chromatography with the washing buffer (50 mM Tris-HCl, pH 8.0, 500 mM NaCl, 25 mM imidazole) and the elution buffer (50 mM Tris-HCl pH 8.0, 500 mM NaCl, 500 mM imidazole). All the proteins were further purified by size-exclusion chromatography (Superdex-200 26/60, GE healthcare) with the buffer containing 25 mM HEPES, pH 7.5, 150 mM NaCl, 1 mM MgCl_2_, 1 mM EGTA, 1 mM DTT for the MD-NC-CC1ab tandem and 25 mM HEPES, pH 7.5, 150 mM NaCl, 1 mM EDTA, 1 mM DTT for the CFCMM supramodule.

### Size-exclusion chromatography coupled with multi-angle light scattering (SEC-MALS)

Protein samples (~1 mg/ml in 25 mM HEPES, pH 7.5, 150 mM NaCl, 1 mM MgCl_2_, 1 mM EGTA, 1 mM DTT) were analyzed with static light scattering by injection of them into an Agilent FPLC system with a SEC protein column for MALS (WTC-030S5, Wyatt Technology). The chromatography system was coupled with an 18-angle light-scattering detector (DAWN HELEOS II, Wyatt Technology) and a differential refractive index detector (Optilab rEx, Wyatt Technology). Masses (molecular weights) were calculated with ASTRA (Wyatt Technology). Bovine serum albumin (Sigma) was used as the calibration standard.

### Crystallization, data collection, and structural determination

Crystals of KLP-6 (∼12 mg/ml) were grown in 0.2 M sodium malonate, pH 7.0, 22% (w/v) PEG3350. All the crystals were obtained by using the sitting-drop vapor-diffusion method at 16 °C. Before being flash-frozen in liquid nitrogen, crystals were soaked in the mother liquor supplemented with 15% (w/v) sucrose for cryoprotection. Diffraction data were collected at the beamline BL19U at the Shanghai Synchrotron Radiation Facility (SSRF) with a wavelength of 0.979 Å at 100 K^32^, and were processed and scaled using HKL2000^33^. The structure of KLP-6 was determined by the molecular replacement method with the structures of the motor domain (PDB code: 6A1Z), the MBS domain (PDB code: 5B64) and the FHA domain (PDB code: 3FM8) from KIF13B as searching models using PHASER^34^. The structure was further fitted and rebuilt with COOT^35^. The residues of CC1, CC2 and the MATH domain were manually modeled into the structure according to the 2Fo-Fc and Fo-Fc electron density maps. The structure was further refined with PHENIX^36^. Due to the poor electron density maps, residues 263–269, 299–302, 432–434, 579–589, and 842–928 were not included in the final structural model. The structure figures were prepared with the program UCSF ChimeraX^37^ and PyMOL (https://pymol.org/2/). The statistics for data collection and structural refinement were summarized in Supplementary Table 1.

### Molecular dynamics simulations

The crystal structure of full-length KLP-6 solved in this work was used as the starting model in the molecular dynamics simulations. Two additional isolated models, the N-terminal MD-NC-CC1ab tandem (D5-K431, ADP, and Mg^2+^) and the C-terminal CFCMM supramodule (A433-R841), were generated by extracting them from the crystal structure of full-length KLP-6. All the models were subsequently solvated in the rectangular water boxes with the TIP3P water model and were neutralized by 150 mM KCl. All the systems were first pre-equilibrated with the following three steps: (1) 10,000 steps energy minimization with the heavy atoms of protein, ADP and Mg^2+^ constrained; (2) 3 × 2 ns equilibration simulations under 1-fs time step with these atoms constrained by 5, 1, and 0.2 kcal/mol/Å^2^ spring sequentially; (3) 10-ns equilibration simulation under 1 fs time step without any constrains. The resulted systems were subjected to productive simulations for ~200 ns with 2-fs time step. All the simulations were performed with NAMD2.14 software^38^ using CHARMM36m force field^39^. The simulations were performed in NPT ensemble (1 atm, 310 K) using a Langevin thermostat and Nosé-Hoover Langevin piston method, respectively. 12 Å cut off with 10–12 Å smooth switching was used for the calculation of the VDW interactions. The electrostatic interactions were computed using the PME method under periodic boundary conditions. The system preparations and illustrations were conducted using VMD^40^.

### Microtubule-stimulated ATPase assay

Measurements of the microtubule-stimulated ATPase activities of the wild-type KLP-6 and various mutants were performed by using the HTS Kinesin ATPase Endpoint Assay Biochem Kit (Cytoskeleton, Inc., BK053). Briefly, all of the measurements were based on the malachite green phosphate assay to probe inorganic phosphate generated during the reaction. A standard curve of phosphate was made to estimate the amount of phosphate generated. Each protein sample had two replicates, and each measurement was repeated at least three times independently. The kinesin-1 heavy chain (KHC) supplied in the kit was used as the control. All of the data were analyzed by using the Microsoft Excel and the GraphPad Prism8 program.

### Cell culture, imaging, and data analysis

The full-length wild-type KLP-6 and various mutants were each cloned into a pEGFP-N3 vector. N2A cells (ATCC, CCL-131) were cultured in DMEM (Gibco, 11965092) containing 10% (v/v) FBS (Gibco, 10099-141) and were grown at 37 °C. The cells were transfected with the wild-type KLP-6 and various mutants by Lipofectamine 3000 (Invitrogen, L3000-015), according to the manufacturer’s instructions. An Olympus FV1000 Laser Scanning Confocal Microscopy was used to obtain fluorescence images with a 60× (NA = 1.42) oil objective and the Olympus FV10-ASW 3.1 imaging software. Confocal settings used for image capture were kept constant in comparison experiments. During the cellular distribution data analysis, the specific regions of the cell body (excluding the nucleus) and the tip of each cell were chosen, and the average fluorescence intensities (FIs) were calculated, respectively. All of the fluorescence images were processed and analyzed by ImageJ (NIH). The final quantification graphs were generated by the GraphPad Prism8 program.

### Thermal denaturation assay

The thermal denaturation analysis of the MD-NC-CC1ab tandem and CFCMM supramodule of KLP-6 and their mutants was performed on a Chirascan Plus (Applied Photo Physics) spectrometer using a 10 mm path-length quartz cell. The protein samples (~0.2 mg/ml) were in the buffer containing 25 mM HEPES, pH 7.5, 150 mM NaCl, 1 mM MgCl_2_, 1 mM EGTA, 1 mM DTT for the MD-NC-CC1ab tandem and in the buffer containing 25 mM HEPES, pH 7.5, 150 mM NaCl, 1 mM EDTA, 1 mM DTT for the CFCMM supramodule. A buffer-only sample was used as the reference. The CD spectra were recorded from 20 to 100 °C at 222 nm by using a 1 °C/min temperature ramp with a 60 s delay. Data analysis was performed using Origin (Origin Lab).

## Supplementary information

Supplementary Information

reporting-summary

## Data Availability

The atomic coordinate of full-length kinesin-3 KLP-6 has been deposited in the Protein Data Bank with the accession code 7WRG. The atomic coordinates used for molecular replacement or structural comparison were downloaded from the Protein Data Bank 6A1Z, 5B64, 3FM8, 6A20, 1I5S, 4HNA, 1D01, and 5DJO. Source data are provided with this paper.

## Source data

Source Data
