# Supplementary material for: The architecture of kinesin-3 KLP-6 reveals a multilevel-lockdown mechanism for autoinhibition
Source: Nat Commun. 2022 Jul 25;13:4281. doi: 10.1038/s41467-022-32048-y (PMC9314371; doi:10.1038/s41467-022-32048-y)
Supplement: Supplementary file 1 — Supplementary Information [file 41467_2022_32048_MOESM1_ESM.pdf]

Supplementary Information

The architecture of kinesin-3 KLP-6 reveals a multilevel-lockdown mechanism for autoinhibition

Wenjuan Wang, Jinqi Ren, Weiye Song et al.

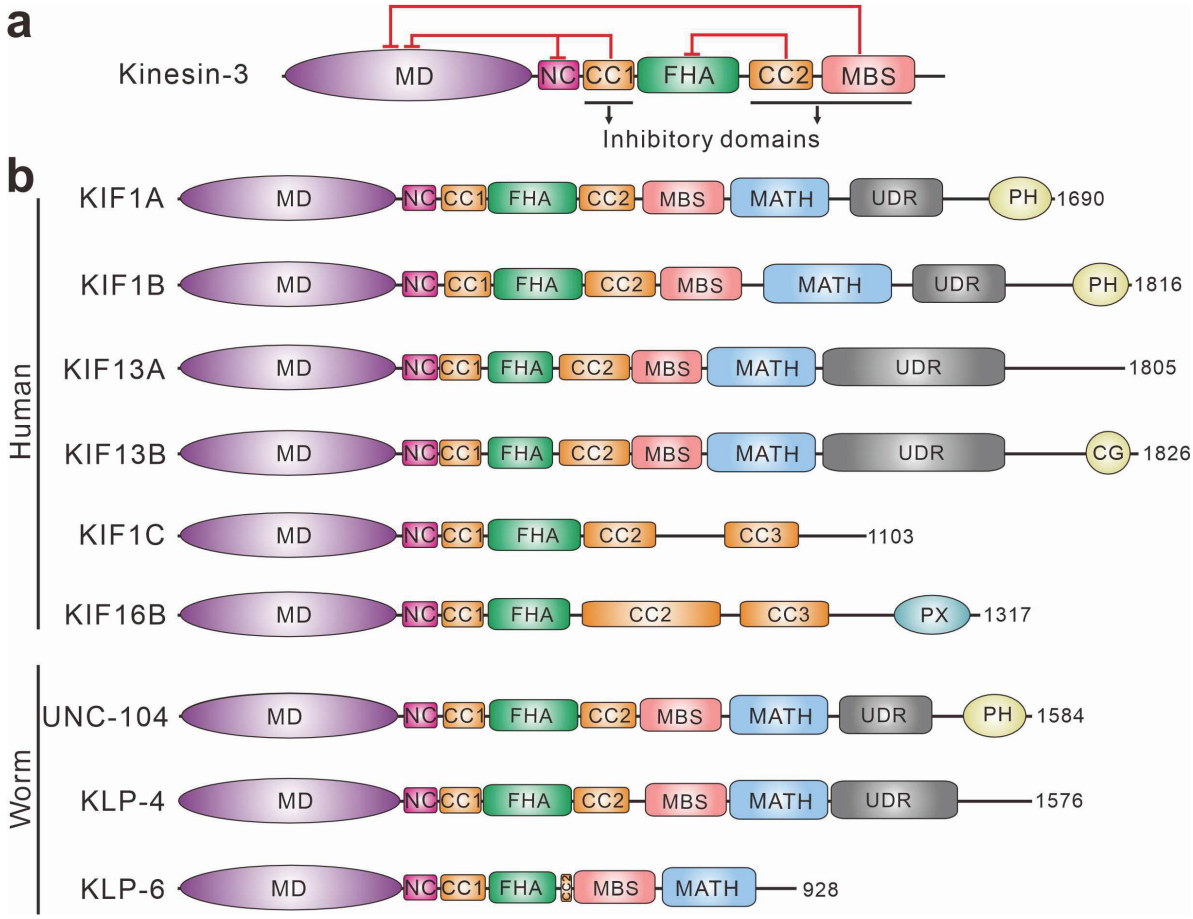

Supplementary Fig. 1 A summary of the domain organizations of kinesin-3 motors from different species. (a) A summary of the intra-molecular contacts in kinesin-3 to maintain the motor in a self-folded conformation for autoinhibition. CC1, CC2 and the MBS domain are the known inhibitory domains in kinesin-3 for autoinhibition. The intra-molecular interaction network mediated by the inhibitory domains is highlighted by red bar-headed arrows. **(b)** Domain organizations of different members of kinesin-3 from human and worm. In human, KIF1A, KIF1B, KIF13A and KIF13B contain a similar set of domains, while some domains such as the MBS and MATH domains are missing in KIF1C and KIF16B. In worm, KLP-6 contains all the known inhibitory domains (including CC1, CC2 and the MBS domain) and the internal loops between the various domains are much shorter.

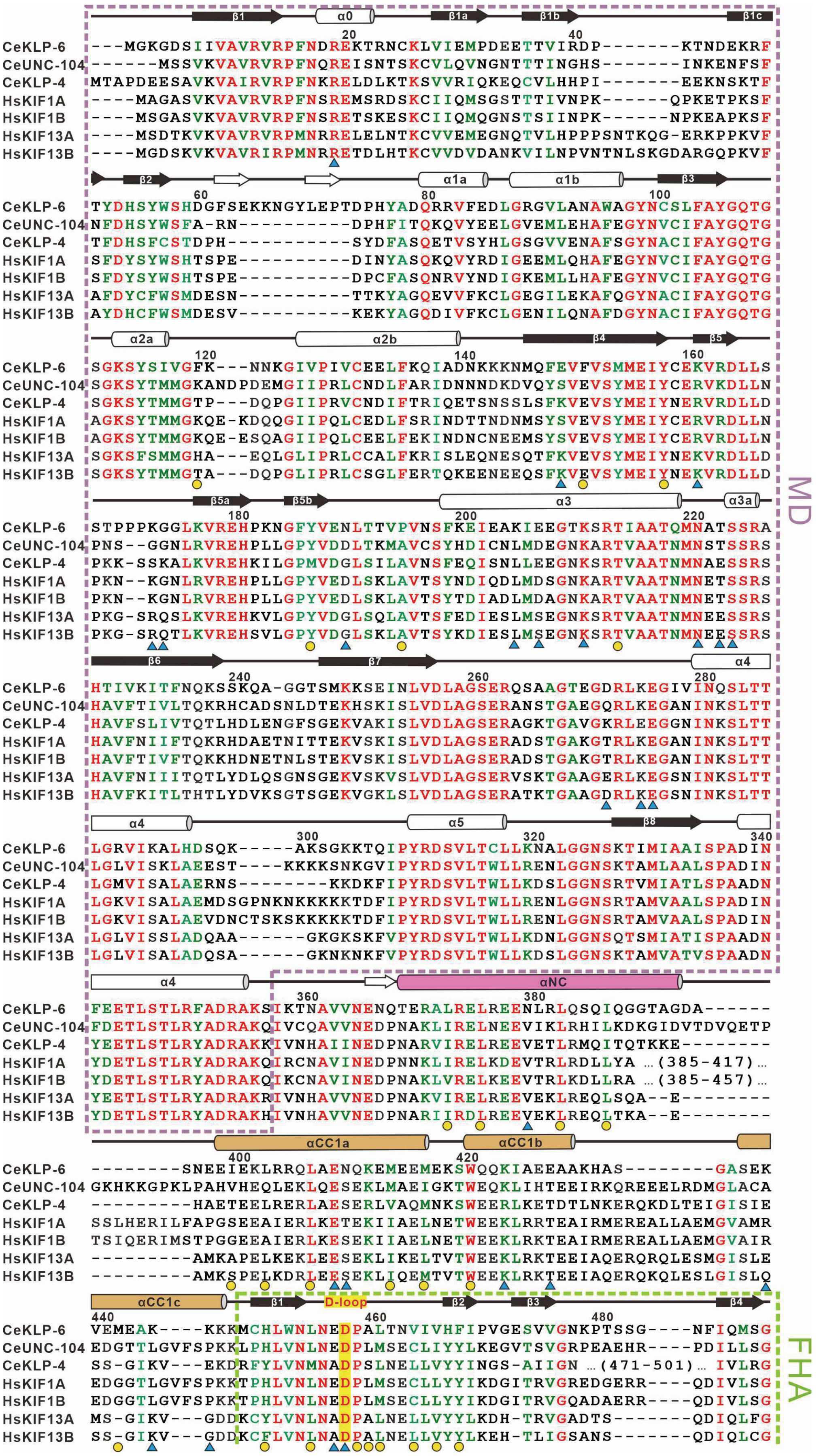
CeKLP-6
CeUNC-104
CeKLP-4
HsKIF1A
HsKIF1B
HsKIF13A
HsKIF13B
MD
FHA
D-loop
αNC
αCC1a
αCC1b
αCC1c

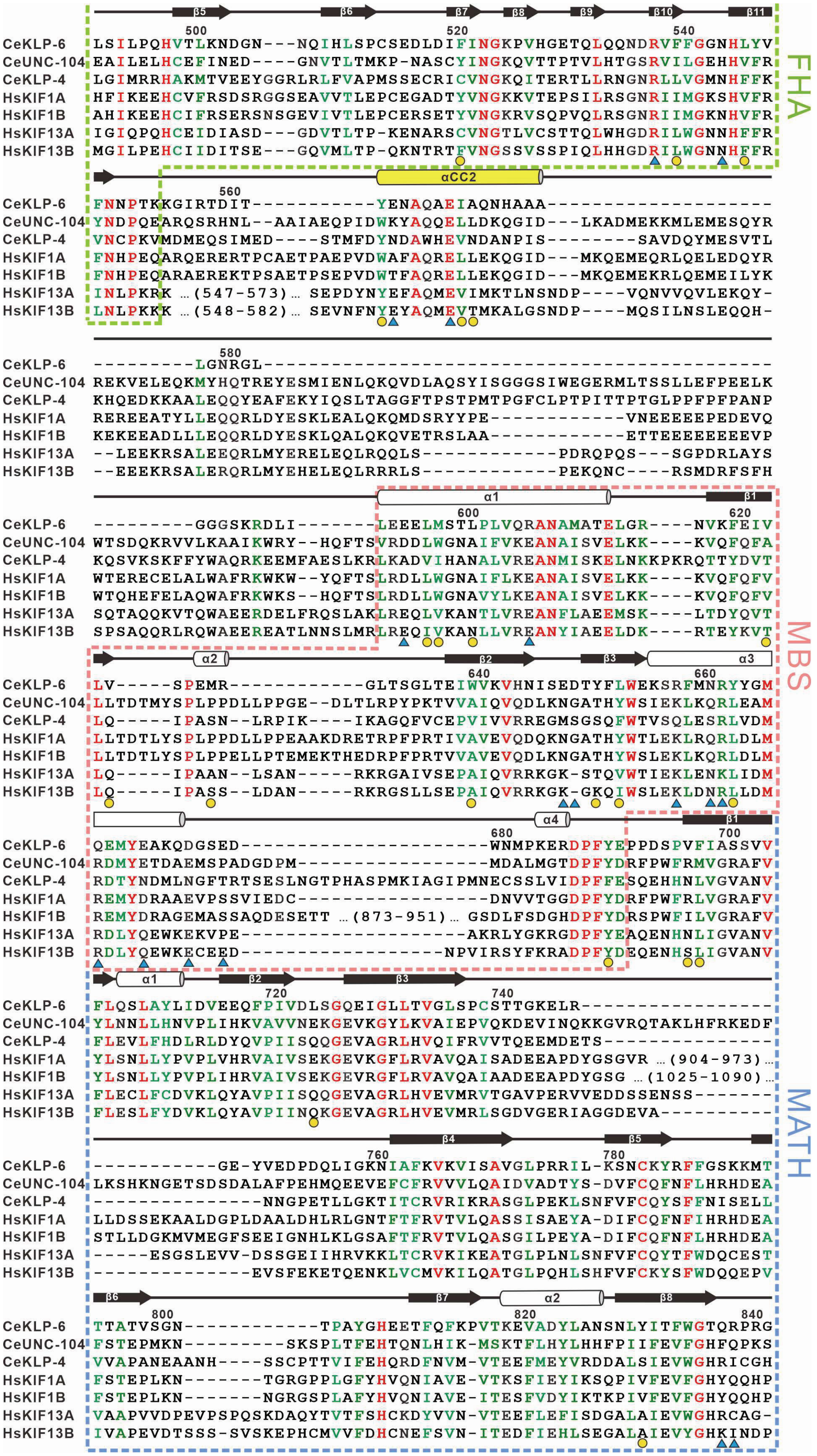

FHA
MBS
MATH
CeKLP-6
CeUNC-104
CeKLP-4
HsKIF1A
HsKIF1B
HsKIF13A
HsKIF13B
β5
β6
β7
β8
β9
β10
β11
αCC2
α1
β1
α2
β2
β3
α3
α4
β4
β5
β6
β7
β8

Supplementary Fig. 2 Structure-based sequence alignment of kinesin-3 motors from different species. Most of the essential domains are highly conserved in kinesin-3 across different species, and the internal loops between the various domains of KLP-6 are much shorter. The identical residues and highly conserved residues are colored in red and green, respectively. The residue numbers and secondary structures of KLP-6 are marked at the top. The different domains of KLP-6 are indicated by dashed boxes with the color scheme in Fig. 1c. The unique D-loop in the FHA domain is highlighted in yellow. The essential residues responsible for the hydrophobic and polar contacts in the inter-domain interfaces are marked with yellow dots and blue triangles, respectively, at the bottom.

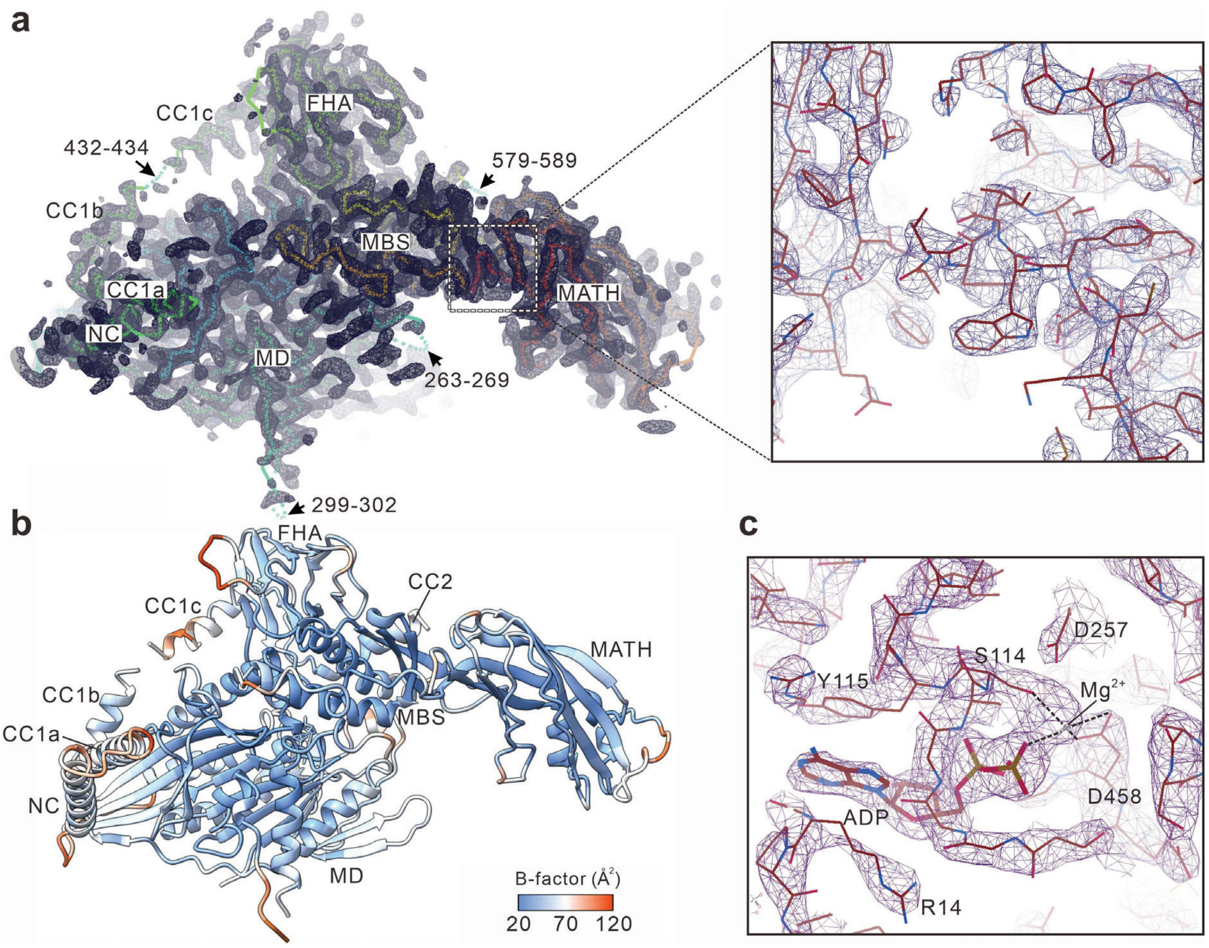

Supplementary Fig. 3 Validation of the crystal structure of full-length KLP-6. (a) The 2mFo-DFc electron density map of the crystal structure of full-length KLP-6 (contoured at 1.5 σ level). The resolved structural parts are shown in the ribbon representation. The inset shows a close-up view of the electron density map of a selected region with the structure shown in the stick representation. Unresolved fragments due to the poor electron density maps are marked by arrows. **(b)** The structural model of full-length KLP-6 colored with the B-factor (according to the value of the B-factor, colored from blue through white to red). **(c)** A close-up view of the electron density maps of ADP and Mg^{2+} in the nucleotide-binding pocket (the 2mFo-DFc electron density map, contoured at 1.5 σ level).

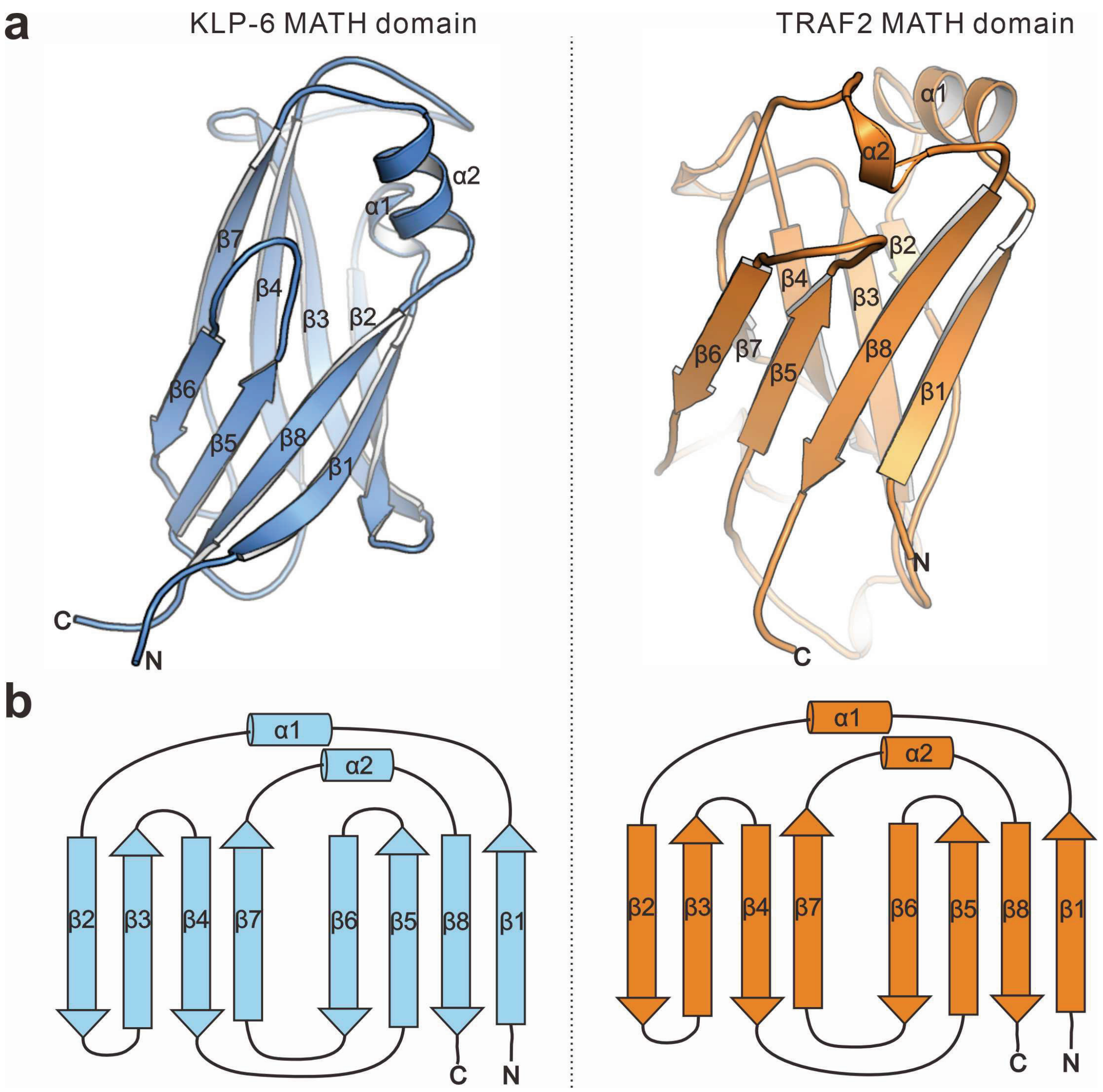

Supplementary Fig. 4 Structural analysis of the newly identified MATH domain of KLP-6. (a) A ribbon diagram of the structures of the MATH domain from KLP-6 and TRAF2 (PDB code: 1D01). The MATH domain of KLP-6 is colored in blue and that of TRAF2 is colored in orange. **(b)** A schematic diagram showing the folding topology of the MATH domain from KLP-6 and TRAF2. The two domains adopt a similar folding topology.

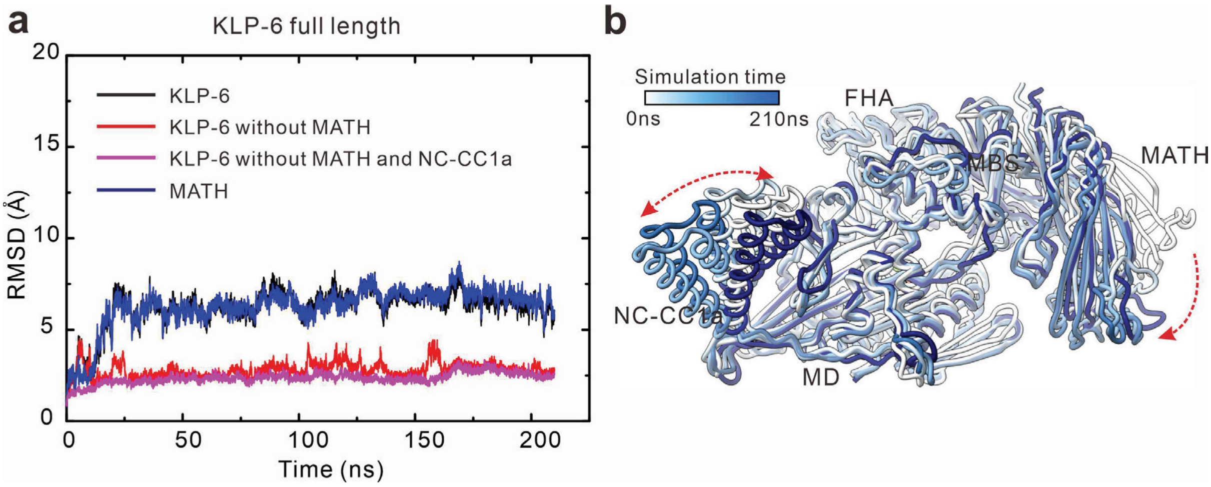

Supplementary Fig. 5 Molecular dynamics simulations of the structure of full-length KLP-6. (a) The average root-mean-square deviation (RMSD) for the backbone atoms in the KLP-6 structure is plotted. The RMSDs of full-length KLP-6, KLP-6 without the MATH domain, KLP-6 without the MATH domain and the NC-CC1a bundle, and the MATH domain alone are colored in black, red, magenta, and blue, respectively. **(b)** A sausage-style cartoon diagram showing the representative snapshots of the KLP-6 structure during the simulations. The central core of the KLP-6 structure is stable, while the NC-CC1a bundle and the MATH domain at the two peripheries show certain flexibilities (indicated by dashed arrows).

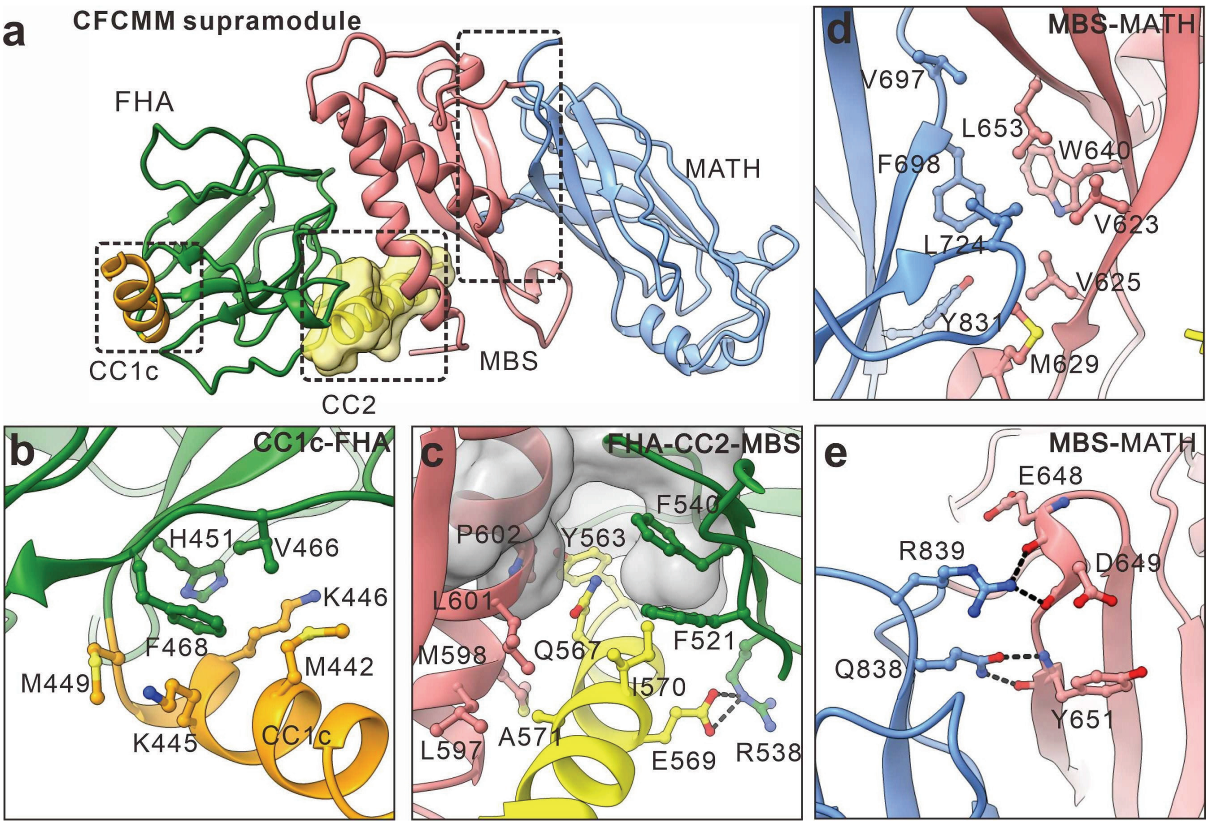

Supplementary Fig. 6 Non-MD-involved inter-domain interfaces for the CFCMM supramodule formation. **(a)** A ribbon diagram of the structure of the CC1c-FHA-CC2-MBS-MATH (CFCMM) supramodule. The color scheme follows that in Fig. 1c. **(b-e)** A combined ribbon-and-stick model showing the non-MD-involved inter-domain interfaces in the CC1c-FHA (b), FHA-CC2-MBS (c) and MBS-MATH sites (d-e). The sidechains of the residues in the inter-domain interfaces are shown as sticks.

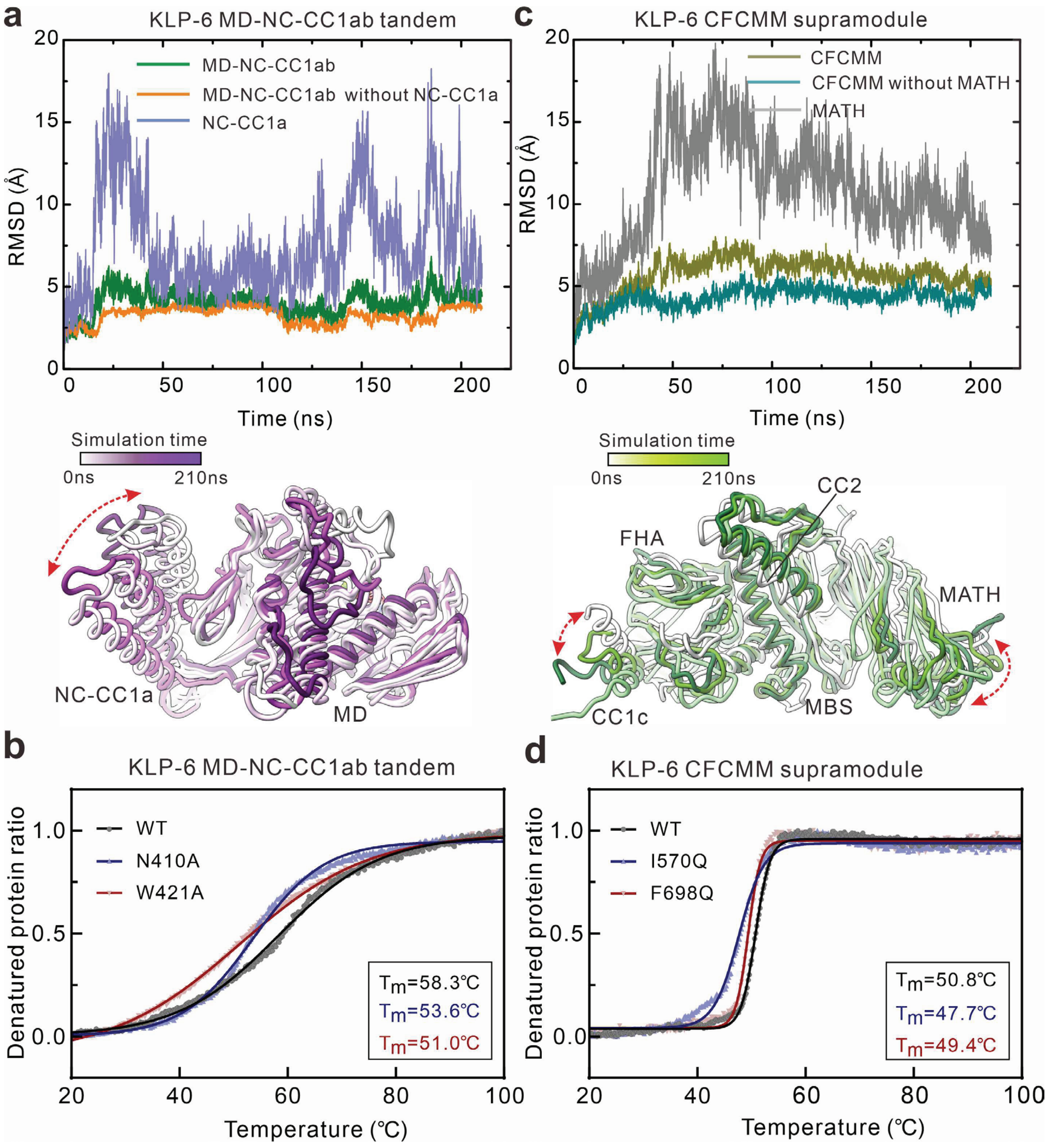

Supplementary Fig. 7 Evaluation of the potential formation of the sub-complexes. (a) Molecular dynamics simulations of the MD-NC-CC1ab tandem (upper panel). The average RMSD for the backbone atoms is plotted. The RMSDs of the MD-NC-CC1ab tandem, the MD-NC-CC1ab tandem without the NC-CC1a bundle and the NC-CC1a bundle alone are colored in green, orange and light purple, respectively. A sausage-style cartoon diagram showing the representative snapshots of the MD-NC-CC1ab tandem during the simulations (lower panel). The NC-CC1a bundle shows certain flexibilities (indicated by dashed arrows). **(b)** CD-based thermal denaturation assay of the MD-NC-CC1ab tandem and its mutants. Tm is the temperature at the half of full denaturation. **(c)** Molecular dynamics simulations of the CFCMM supramodule (upper panel). The average RMSD for the backbone atoms is plotted. The RMSDs of the CFCMM supramodule, the CFCMM supramodule without the MATH domain and the MATH domain alone are colored in brown, dark cyan and grey, respectively.

A sausage-style cartoon diagram showing the representative snapshots of the CFCMM supramodule tandem during the simulations (lower panel). The short CC1c and MATH domain show certain flexibilities (indicated by dashed arrows). **(d)** CD-based thermal denaturation assay of the CFCMM supramodule and its mutants. Tm is the temperature at the half of full denaturation.

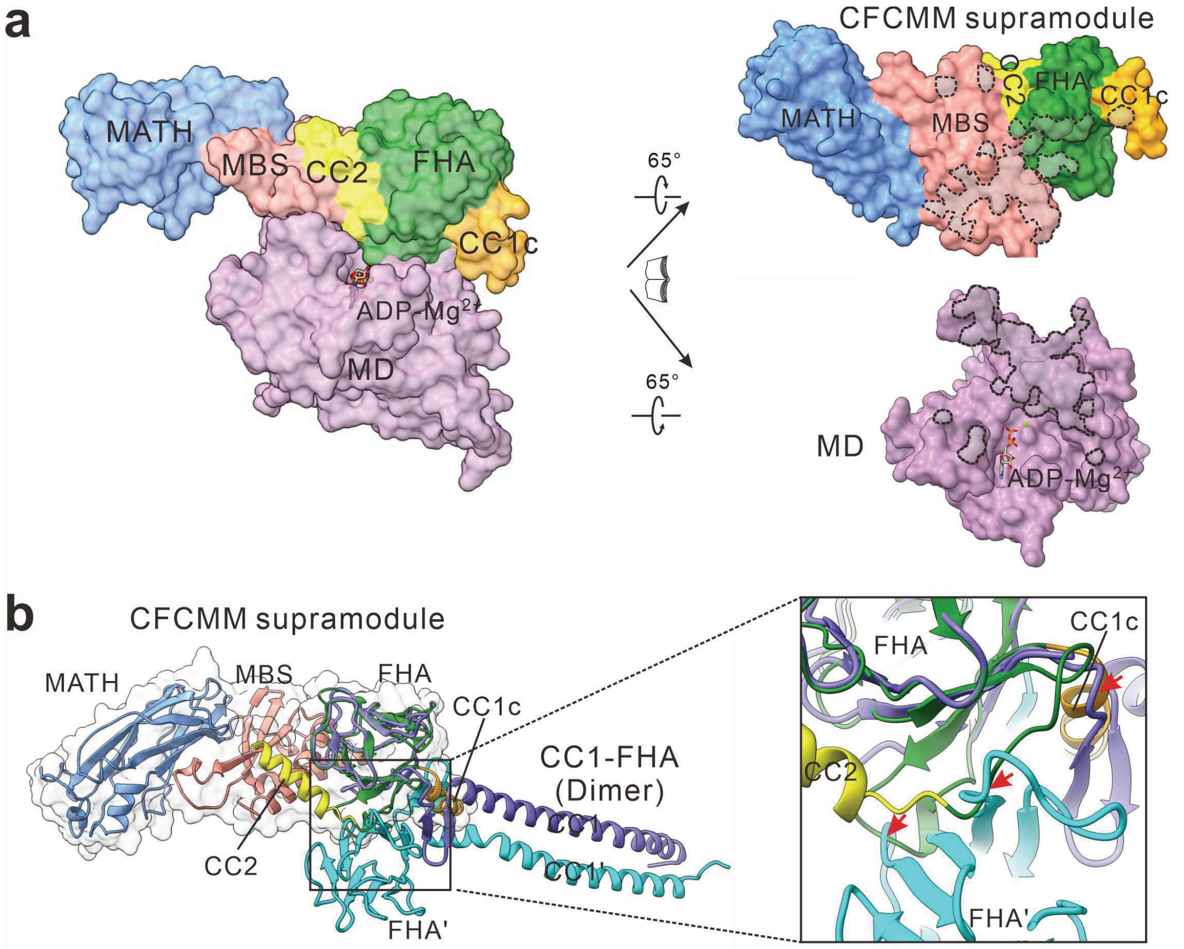

Supplementary Fig. 8 Integration of the CFCMM supramodule by CC2 for autoinhibition. (a) An open-book view of the binding interfaces between the CFCMM supramodule and MD. The CC2 helix integrates the FHA, MBS and MATH domains to form the CFCMM supramodule that wraps around the MD for autoinhibition. **(b)** Structural comparison of the CFCMM supramodule from KLP-6 with the CC1-FHA dimer from KIF13A (PDB code: 5DJO). Upon superimposition of the FHA domain, the steric clashes between the CFCMM supramodule and the CC1-FHA dimer appear (highlighted by red arrows). The CFCMM supramodule of KLP-6 is in the surface representation, and the CC1-FHA dimer of KIF13A is in the ribbon representation.

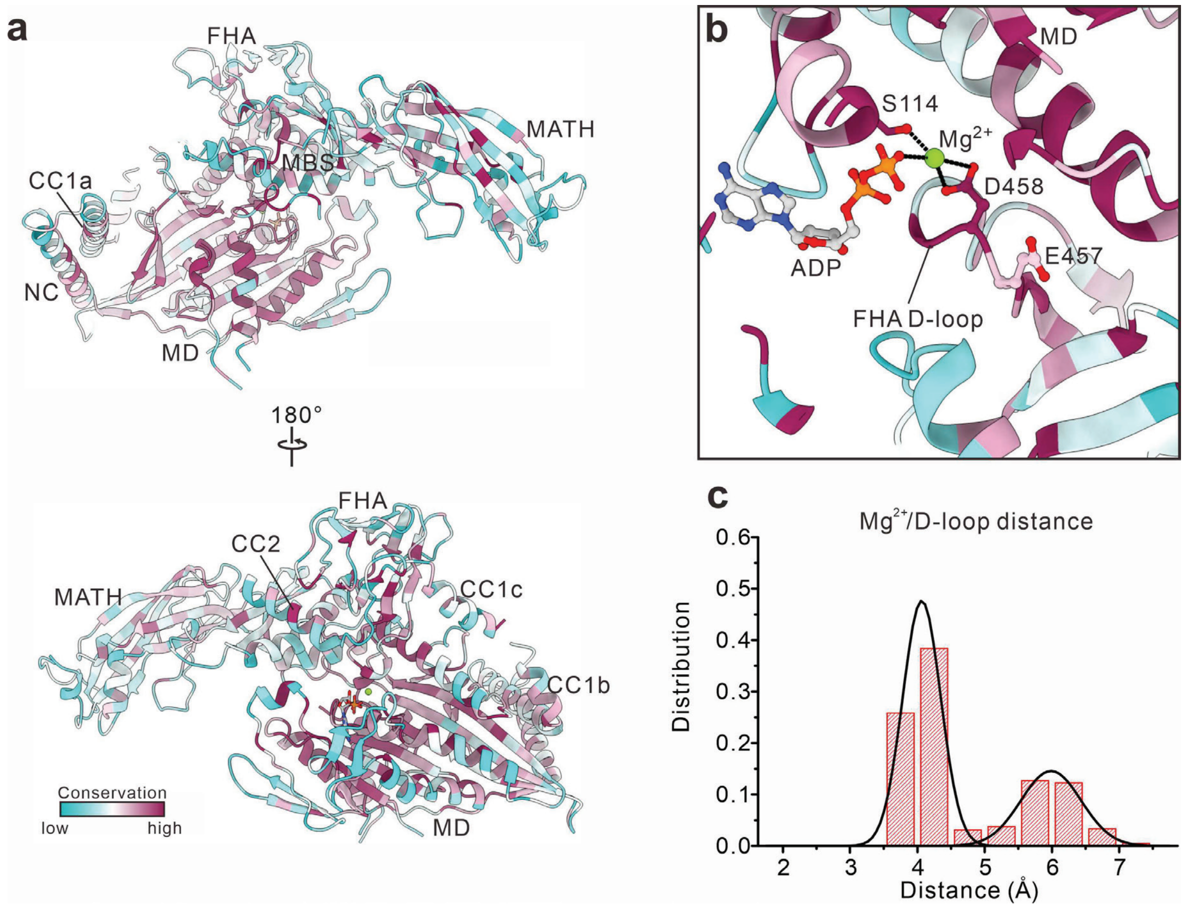

Supplementary Fig. 9 Structural conservation analysis of KLP-6. (a) A ribbon diagram of the structure of KLP-6 colored by the sequence conservation (from the low conservation to high conservation, colored from cyan through white to purple). **(b)** A close-up view of the D-loop of the FHA domain showing the high conservation of D458 for coordinating Mg^{2+}. **(c)** Distribution of the distance between Mg^{2+} and D458 in the D-loop of the FHA domain during the molecular dynamics simulations of full-length KLP-6. Source data are provided as a Source Data file.

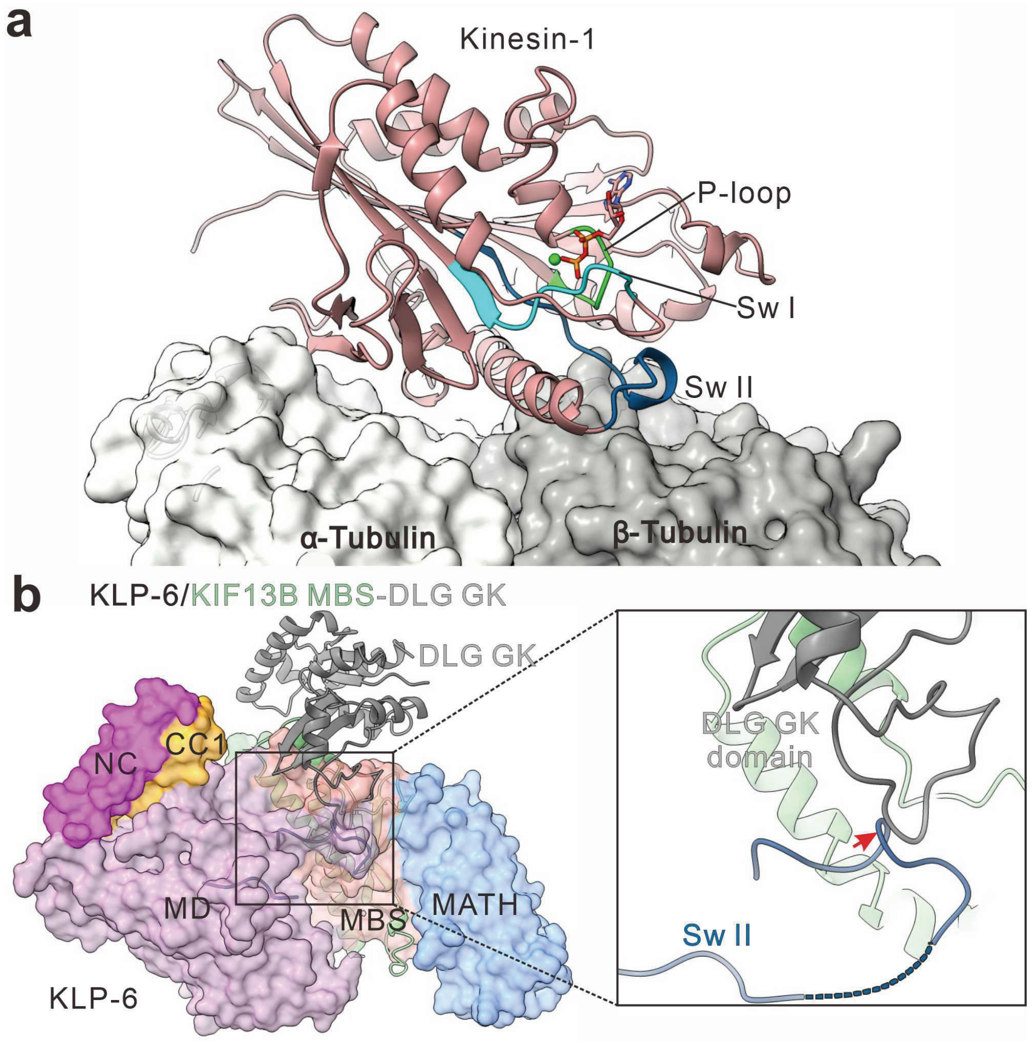

Supplementary Fig. 10 Potential role of the MBS domain for the motor activation by binding of the GK domain. (a) A ribbon diagram of the structure of the kinesin-1/tubulin complex (PDB code: 4HNA). In this complex structure, Switch II of the MD is neighboring to the microtubule-binding site for coupling the binding of microtubules with the ATPase activity of the MD. ADP in the nucleotide-binding pocket is in the stick representation and Switch I/II are colored in cyan and navy blue, respectively. The P-loop and Mg^{2+} are highlighted in green. **(b)** Structural comparison of KLP-6 with the MBS/GK complex formed between KIF13B and DLG (PDB code: 5B64). In KLP-6, most of the GK-binding site of the MBS domain is exposed and accessible for binding to the GK domain. Upon superimposition of the MBS domain, the clashes between the GK domain and Switch II indicate that the binding of the GK domain would somewhat impact the autoinhibited conformation and might contribute to the motor activation. KLP-6 is in the surface representation, and the MBS/GK complex (colored in light green and grey, respectively) is in the ribbon representation. The structural clashes between the GK domain and Switch II of the MD are highlighted in a closed-up view and indicated by red arrows.

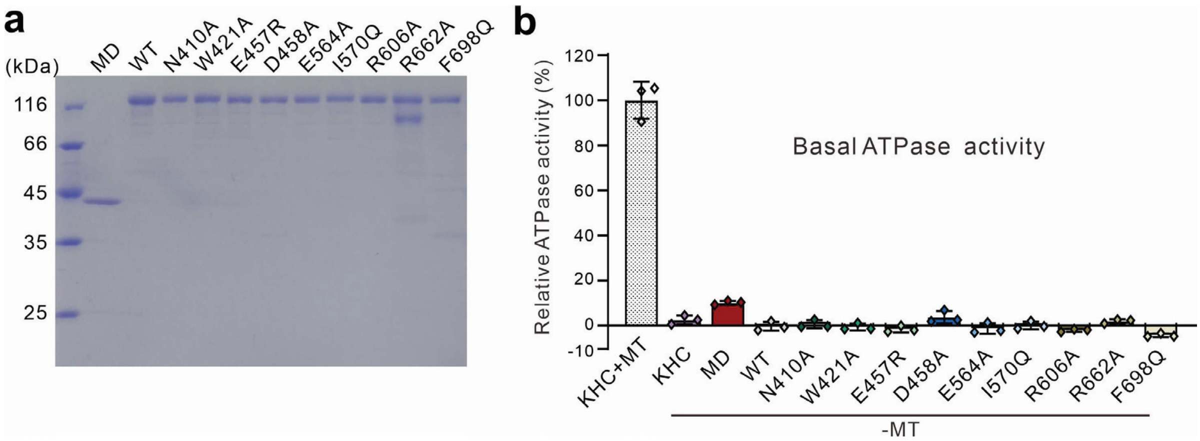

Supplementary Fig. 11 Basal ATPase activities of KLP-6 and its mutants without microtubules. (a) The SDS-PAGE analysis of the protein qualities of KLP-6 and its mutants used for the ATPase activity assay. The experiment was repeated three times with similar results. Source data are provided as a Source Data file. **(b)** Basal ATPase activities of KLP-6 and its mutants. The microtubule-stimulated ATPase activity of KHC was used as the calibration standard, and the data were normalized by the microtubule-stimulated ATPase activity of KHC as 100%. Each protein sample had two replicates and each measurement was repeated three times independently. Each bar represents the mean value ± SD. Source data are provided as a Source Data file.

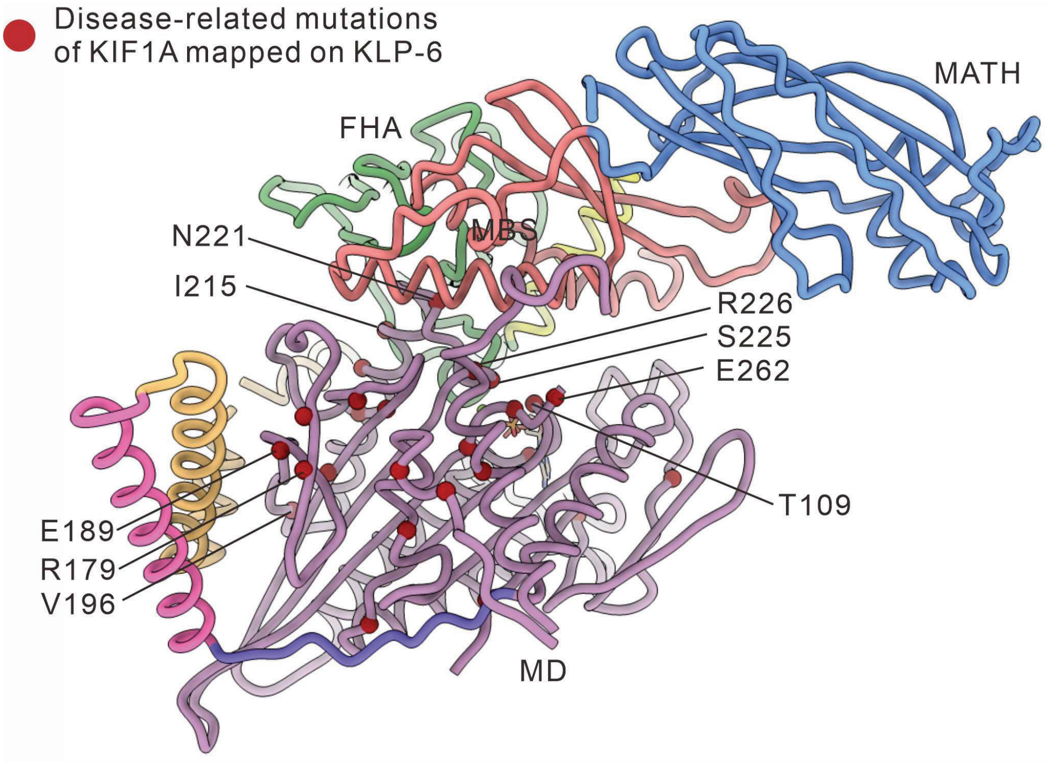

Supplementary Fig. 12 A combined cartoon and sphere representation showing the disease-related mutations. Based on the sequence alignment of KIF1A and KLP-6, the disease-related mutations in KIF1A are mapped onto the self-folded compact structure of KLP-6 and shown as red spheres. The residues (with the disease-related mutations) that are located in the inter-domain interfaces are highlighted and labeled.

Supplementary Table 1 Data collection and structural refinement statistics

A. Diffraction data	KLP-6
Space group	$P2_1$
Wavelength (Å)	0.979
Cell dimensions	
a, b, c (Å)	56.1, 68.8, 253.2
α, β, γ (°)	90,92.7,90
Resolution (Å)	50.0-3.2(3.31-3.20)[a]
Unique reflections	32692(3292)
I/σ(I)	9.3(2.7)
Multiplicity	6.6(6.6)
Completeness (%)	98.9(99.6)
B. Refinement	
Rwork(%)	21.3(27.7)
Rfree(%)	26.3(30.6)
Mean B factors (Å^2)	71.0
R.m.s. deviation[b]	
Bond length (Å)	0.002
Bond angles (°)	0.548
Ramachandran plot(%)	
Favored region	94.0
Allowed region	6.0
Disallowed region	0

[a]The values in parentheses refer to the highest resolution shell.

[b]Root mean square deviation from ideal values.

Supplementary Table 2 Primers used in this study

Primer	Sequence(5’-3’)
pFastBac1-Xho1-KLP-6-1F	ctagagcctgcagtctcgagatgggaaagggtg
pFastBac1-Kpn1-KLP-6-928R	gaacttccagaagcttggtacctttcgcctttgg
pEGFPN3-KLP-6-1F	ctcgagctcaagcttcgaattcgccaccatgggaaagggtgactccata
pEGFPN3-KLP-6-928R	gatcccgggcccgcggtacctttcgcctttggtttcttcgat
pETM3C-Nde1-KLP-6-1F	gaaggagatatacatatgggaaagggtgactccataatc
pETM3C-Xho1-KLP-6-370R	gtggtggtggtggtgctcgagttctgtttgattctcg
pETM3C-Xho1-KLP-6-434R	gtggtggtggtggtgctcgagactggcatgtttggc
pETM3C-Nde1-KLP-6-435F	gaaggagatatacatatgggagcaagtgaaaaagtg
pETM3C-Xho1-KLP-6-928R	gtggtggtgctcgagtttcgcctttggtttcttcgatcc
N410A_F	gaaacttcgacgccaactggctgaagctcaaaaagaaatggaagaa
N410A_R	ttcttccatttctttttgagcttcagccagttggcgtcgaagtttc
W421A_F	tggaagaaatggagaaatccgcgcaacagaaaatcgcagaag
W421A_R	cttctgcgattttctgttgcgcggatttctccatttcttcca
E457R_F	agaagaagaaaatgtgtcatttatggaatctgaatagagatcctgcattgacaaa
E457R_R	tttgtcaatgcaggatctctattcagattccataaatgacacattttcttcttct
D458A_F	tcatttatggaatctgaatgaagctcctgcattgacaaatgtcatt
D458A_R	aatgacatttgtcaatgcaggagcttcattcagattccataaatga
E564A_F	tacgaactgatatcacttatgcgaatgctcaagctgaaattgc
E564A_R	gcaatttcagcttgagcattcgcataagtgatatcagttcgta
I570Q_F	cttatgagaatgctcaagctgaacaggcacaaaatcatgccgcagct
I570Q_R	agctgcggcatgattttgtgcctgttcagcttgagcattctcataag
R606A_F	caacacttccattagttcaggcagctaatgcaatggccactg
R606A_R	cagtggccattgcattagctgcctgaactaatggaagtgttg
R662A_F	ctttctttgggaaaaatctcgattcatgaatgcttactacggaatgcaag
R662A_R	cttgcattccgtagtaagcattcatgaatcgagatttttcccaaagaaag
F698Q_F	gaaccacctgattccccagtgcagattgcgtcttctgtcgtattt
F698Q_R	aaatacgacagaagacgcaatctgcactggggaatcaggtggttc

Supplementary Table 3 Plasmids and strains used in this study

Plasmids/Strains	Source
pFastBac1-KLP-6-full length	this study
pFastBac1-KLP-6-full length N410A	this study
pFastBac1-KLP-6-full length W421A	this study
pFastBac1-KLP-6-full length E457R	this study
pFastBac1-KLP-6-full length D458A	this study
pFastBac1-KLP-6-full length E564A	this study
pFastBac1-KLP-6-full length I570Q	this study
pFastBac1-KLP-6-full length R606A	this study
pFastBac1-KLP-6-full length R662A	this study
pFastBac1-KLP-6-full length F698Q	this study
pEGFPN3-KLP-6-full length	this study
pEGFPN3-KLP-6-full length N410A	this study
pEGFPN3-KLP-6-full length W421A	this study
pEGFPN3-KLP-6-full length E457R	this study
pEGFPN3-KLP-6-full length D458A	this study
pEGFPN3-KLP-6-full length E564A	this study
pEGFPN3-KLP-6-full length I570Q	this study
pEGFPN3-KLP-6-full length R606A	this study
pEGFPN3-KLP-6-full length R662A	this study
pEGFPN3-KLP-6-full length F698Q	this study
pETM3C-KLP-6-MD	this study
pETM3C-KLP-6-MD-NC-CC1ab	this study
pETM3C-KLP-6-MD-NC-CC1ab N410A	this study
pETM3C-KLP-6-MD-NC-CC1ab W421A	this study
pETM3C-KLP-6-CFCMM	this study
pETM3C-KLP-6-CFCMM I570Q	this study
pETM3C-KLP-6-CFCMM F698Q	this study
Escherichia coli BL21 (codon plus)	lab storage
sf9 cells	Gibco
Escherichia coli DH10Bac	Gibco
N2A cells	ATCC
