# Supplementary material for: The architecture of kinesin-3 KLP-6 reveals a multilevel-lockdown mechanism for autoinhibition
Source: Nat Commun. 2022 Jul 25;13:4281. doi: 10.1038/s41467-022-32048-y (PMC9314371; doi:10.1038/s41467-022-32048-y)
Supplement: Supplementary file 2 — reporting-summary [file 41467_2022_32048_MOESM2_ESM.pdf]

# nature portfolio

Corresponding author(s): Wei Feng

Last updated by author(s): Jul 6, 2022

# Reporting Summary

Nature Portfolio wishes to improve the reproducibility of the work that we publish. This form provides structure for consistency and transparency in reporting. For further information on Nature Portfolio policies, see our Editorial Policies and the Editorial Policy Checklist.

## Statistics

For all statistical analyses, confirm that the following items are present in the figure legend, table legend, main text, or Methods section.

| n/a | Confirmed | |
|---|---|---|
| ☐ | ☒ | The exact sample size (*n*) for each experimental group/condition, given as a discrete number and unit of measurement |
| ☐ | ☒ | A statement on whether measurements were taken from distinct samples or whether the same sample was measured repeatedly |
| ☐ | ☒ | The statistical test(s) used AND whether they are one- or two-sided<br>*Only common tests should be described solely by name; describe more complex techniques in the Methods section.* |
| ☒ | ☐ | A description of all covariates tested |
| ☒ | ☐ | A description of any assumptions or corrections, such as tests of normality and adjustment for multiple comparisons |
| ☐ | ☒ | A full description of the statistical parameters including central tendency (e.g. means) or other basic estimates (e.g. regression coefficient) AND variation (e.g. standard deviation) or associated estimates of uncertainty (e.g. confidence intervals) |
| ☐ | ☒ | For null hypothesis testing, the test statistic (e.g. *F*, *t*, *r*) with confidence intervals, effect sizes, degrees of freedom and *P* value noted<br>*Give P values as exact values whenever suitable.* |
| ☒ | ☐ | For Bayesian analysis, information on the choice of priors and Markov chain Monte Carlo settings |
| ☒ | ☐ | For hierarchical and complex designs, identification of the appropriate level for tests and full reporting of outcomes |
| ☒ | ☐ | Estimates of effect sizes (e.g. Cohen's *d*, Pearson's *r*), indicating how they were calculated |

*Our web collection on statistics for biologists contains articles on many of the points above.*

## Software and code

Policy information about availability of computer code

| | |
|---|---|
| Data collection | Fluorescence images collection: Olympus FV10-ASW 3.1 imaging software. |
| Data analysis | X-ray data process: HKL2000 version 720. Structural determination: PHASER version 2.8.3. Structural model building: COOT version 0.8.9.2. Structural refinement: PHENIX version 1.17. Figure preparation: PyMol version 1.7.0.0 and UCSF ChimeraX version 1.3. Statistic analysis: Microsoft Excel version 2010, GraphPad Prism version 8.0.2, Origin 8.0 (Origin Lab). Molecular dynamics simulations software: NAMD version 2.14, VMD version 1.9.3. |

For manuscripts utilizing custom algorithms or software that are central to the research but not yet described in published literature, software must be made available to editors and reviewers. We strongly encourage code deposition in a community repository (e.g. GitHub). See the Nature Portfolio guidelines for submitting code & software for further information.

## Data

Policy information about availability of data

All manuscripts must include a data availability statement. This statement should provide the following information, where applicable:

- Accession codes, unique identifiers, or web links for publicly available datasets
- A description of any restrictions on data availability
- For clinical datasets or third party data, please ensure that the statement adheres to our policy

The structural factors and atomic coordinate reported in this paper have been deposited in the Protein Data Bank with the accession code 7WRG [https://dx.doi.org/10.2210/pdb7wrg/pdb]. Other protein coordinates used for molecular replacement (PDB: 6A1Z [https://dx.doi.org/10.2210/pdb6a1z/pdb], PDB: 5B64 [https://dx.doi.org/10.2210/pdb5b64/pdb], PDB: 3FM8 [https://dx.doi.org/10.2210/pdb3fm8/pdb]) or structural comparison (PDB: 6A20 [https://dx.doi.org/10.2210/pdb6a20/pdb], PDB: 1I5S [https://dx.doi.org/10.2210/pdb1i5s/pdb], PDB: 4HNA [https://dx.doi.org/10.2210/pdb4hna/pdb], PDB: 1D01 [https://

dx.doi.org/10.2210/pdb1d01/pdb], PDB: 5DJO [https://dx.doi.org/10.2210/pdb5djo/pdb]) are all publicly accessible.
The source data underlying Fig. 4a, Fig. 4c, Supplementary Fig. 9c, Supplementary Fig. 11a, and Supplementary Fig. 11b are provided as a Source Data file.

# Field-specific reporting

Please select the one below that is the best fit for your research. If you are not sure, read the appropriate sections before making your selection.

☒ Life sciences ☐ Behavioural & social sciences ☐ Ecological, evolutionary & environmental sciences

For a reference copy of the document with all sections, see nature.com/documents/nr-reporting-summary-flat.pdf

# Life sciences study design

All studies must disclose on these points even when the disclosure is negative.

| | |
|---|---|
| Sample size | No sample size calculation was performed. For the cellular experiments, the sample size was determined based on common sizes used in previous publications with similar methodologies (for example: DOI: 10.1016/j.str.2012.07.002; DOI: 10.1074/jbc.M115.689091; DOI: 10.1073/pnas.1811209115). For the microtubule-stimulated ATPase assay, 2-3 biological replicates were typical and widely accepted. |
| Data exclusions | No data was excluded from the study. |
| Replication | The experiments were repeated at least twice and all attempts at replication were successful. |
| Randomization | Additional randomization was not performed due to that the experiments were performed simultaneously and in parallel with controls. |
| Blinding | Blinding was not applicable in this study due to the necessity of maintaining sample labeling for analysis between groups. |

# Reporting for specific materials, systems and methods

We require information from authors about some types of materials, experimental systems and methods used in many studies. Here, indicate whether each material, system or method listed is relevant to your study. If you are not sure if a list item applies to your research, read the appropriate section before selecting a response.

## Materials & experimental systems

| n/a | Involved in the study | |
|---|---|---|
| ☒ | ☐ | Antibodies |
| ☐ | ☒ | Eukaryotic cell lines |
| ☒ | ☐ | Palaeontology and archaeology |
| ☒ | ☐ | Animals and other organisms |
| ☒ | ☐ | Human research participants |
| ☒ | ☐ | Clinical data |
| ☒ | ☐ | Dual use research of concern |

## Methods

| n/a | Involved in the study | |
|---|---|---|
| ☒ | ☐ | ChIP-seq |
| ☒ | ☐ | Flow cytometry |
| ☒ | ☐ | MRI-based neuroimaging |

## Eukaryotic cell lines

Policy information about cell lines

| | |
|---|---|
| Cell line source(s) | N2A cells were purchased from American Type Culture Collection (ATCC). Sf9 cells were purchased from Gibco. |
| Authentication | The N2A cells and sf9 cells were authenticated by morphology. |
| Mycoplasma contamination | The cell lines used in this study were tested negative for mycoplasma contamination. |
| Commonly misidentified lines (See ICLAC register) | None of commonly misidentified lines were used in this study. |
